# Triclosan antimicrobial polymers

**DOI:** 10.3934/molsci.2016.1.88

**Published:** 2016-03-29

**Authors:** Richard C. Petersen

**Affiliations:** Department of Biomaterials and Restorative Sciences, University of Alabama at Birmingham, Birmingham, AL, USA

**Keywords:** Antimicrobial, computational chemistry, mechanomolecular, bond rotation, bond entanglements, secondary bonding, polymer, strength, toughness, viscosity

## Abstract

Triclosan antimicrobial molecular fluctuating energies of nonbonding electron pairs for the oxygen atom by ether bond rotations are reviewed with conformational computational chemistry analyses. Subsequent understanding of triclosan alternating ether bond rotations is able to help explain several material properties in Polymer Science. Unique bond rotation entanglements between triclosan and the polymer chains increase both the mechanical properties of polymer toughness and strength that are enhanced even better through secondary bonding relationships. Further, polymer blend compatibilization is considered due to similar molecular relationships and polarities. With compatibilization of triclosan in polymers a more uniform stability for nonpolar triclosan in the polymer solid state is retained by the antimicrobial for extremely low release with minimum solubility into aqueous solution. As a result, triclosan is projected for long extended lifetimes as an antimicrobial polymer additive. Further, triclosan rapid alternating ether bond rotations disrupt secondary bonding between chain monomers in the resin state to reduce viscosity and enhance polymer blending. Thus, triclosan is considered for a polymer additive with multiple properties to be an antimicrobial with additional benefits as a nonpolar toughening agent and a hydrophobic wetting agent. The triclosan material relationships with alternating ether bond rotations are described through a complete different form of medium by comparisons with known antimicrobial properties that upset bacterial cell membranes through rapid fluctuating mechanomolecular energies. Also, triclosan bond entanglements with secondary bonding can produce structural defects in weak bacterial lipid membranes requiring pliability that can then interfere with cell division. Regarding applications with polymers, triclosan can be incorporated by mixing into a resin system before cure, melt mixed with thermoplastic polymers that set on cooling into a solid or alternatively applied as a coating through several different methods with dissolving into an organic solvent and dried on by evaporation as a common means.

## 1. Introduction

**Figure 1:**
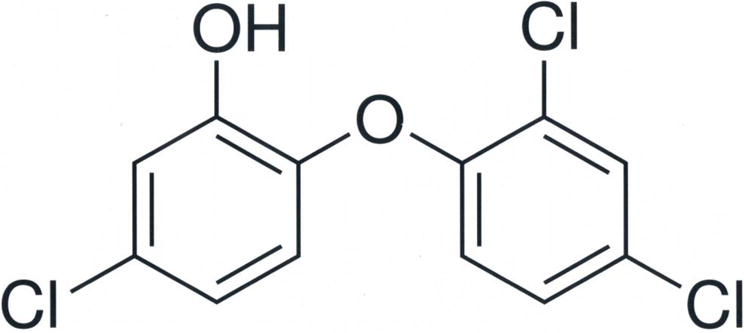
Triclosan molecule depicted as a planar molecule with no ether oxygen bond rotation.

Triclosan is a trichlorinated diphenyl ether antimicrobial with one hydroxyl group, Figure 1. Triclosan has broad-spectrum activity at low concentrations to inhibit both gram positive and gram negative bacteria and also many virus and fungus [1,2]. Triclosan exists as a white crystalline powder with a melting point of 54–57 °C and decomposition temperature greater than 280 °C [1–3]. Triclosan is nonpolar as a sparingly soluble molecule in water at 0.001 grams/100 grams water (10^−5^ g/mL) [1–3] and soluble in most organic solvents [1–3]. Nonpolar properties of triclosan are reflected in a solubility of 40 grams/100 grams oleic acid [1] that is a common fatty acid as a constituent of phospholipid cell membranes [4]. Further, triclosan resists hydrolysis and is very stable in acids [1].

Because triclosan is a nonpolar molecule the majority of chemical interactions result from secondary bonding available through the ether oxygen and phenyl hydroxyl functional groups. In terms of application, triclosan is the most studied antimicrobial concerning bacterial resistance [2]. Of significant importance, no epidemiological data exists demonstrating any association between triclosan and bacterial resistance in humans [2]. Triclosan has been used as an antimicrobial for almost 50 years clinically and in consumer products such as cosmetics or toothpaste and plastics [2,3]. Triclosan has multiple bacterial target sites for damage depending on concentrations [2]. Triclosan is bacteriostatic to prevent microbes from growing at low concentrations by inhibiting an enzyme involved in fatty acid synthesis [2,3]. On the other hand, triclosan is bactericidal to kill microbes directly at higher concentrations by destabilizing bacterial membranes and also by introducing intercalating defects into a bacterial membrane [2].

## 2. Computational chemistry conformational analysis

**Figure 2:**
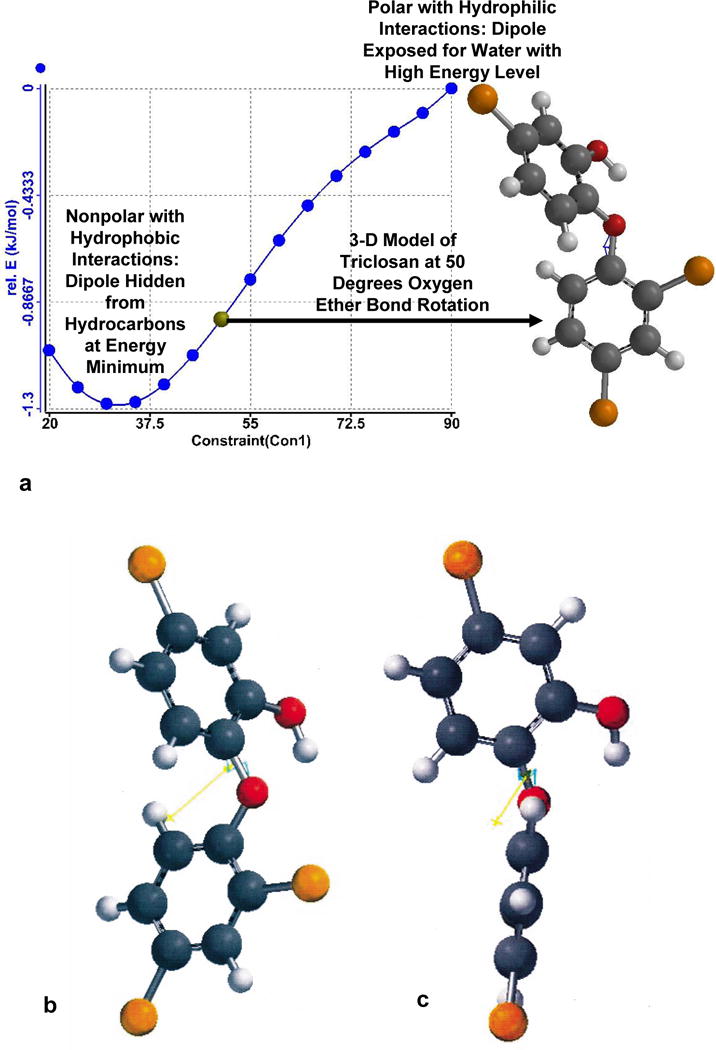
(a) Computational energy profile with rotations of the ether oxygen bonds from 20° through 90° in 5 degree increments to include a 3D model of Triclosan at a 50° bond rotation. (b) 3D model with energy minimum at approximately a 30° bond rotation. (c) 3D model with energy maximum at a 90° bond rotation. Yellow arrows in (b) and (c) depict directions for the dipoles near the ether oxygen atom roughly toward the hydroxyl oxygen atom. 3D models as colors for atoms include: Oxygen-Red; Carbon-Grey; Hydrogen-White; Chlorine-Orange.

The energy profile for the triclosan molecule oxygen ether bond rotation has been calculated by computational chemistry method with Spartan Software from Wavefunction, Newport Beach, CA and plotted from 20° to 90°, Figure 2(a) with 3D molecular structure at a 50° rotation. Computational restricted Hartree-Fock self-consistent field (SCF) method calculation was performed using Pulay DIIS extrapolation. Molecular conformations for bond rotations tend to hide nonbonding lone pair electron dipoles in nonpolar or hydrophobic environments and are expected to expose electron dipoles in polar or hydrophilic environments [5,6]. The energy minimum for triclosan occurs in hydrophobic environments similar to a cell membrane at about 30 degrees bond rotation of the aromatic rings through the center ether oxygen bonds, Figure 2(a) and 2(b). The energy minimum is not at 0.0 degrees in part because intramolecular hydrogen bonding is considered between the hydroxyl group with both the opposing chlorine group on the opposite aromatic ring and also with the ether oxygen that together are thought to twist the triclosan molecule into a skewed conformation [7]. However, most likely steric contact by hydrogen atoms from opposite triclosan aromatic rings creates repulsive forces at 0.0 degrees ether bond rotation [8]. On the other hand the energy maximum occurs in hydrophilic environments similar to biologic fluids at 90 degrees bond rotation of the aromatic rings through the center ether oxygen bonds [8], Figure 2(c). As a consequence triclosan has mechanomolecular capability to fluctuate rapidly where different polar environments exist particularly accentuated at a cell membrane and biologic fluid interface [8].

**Figure 3:**
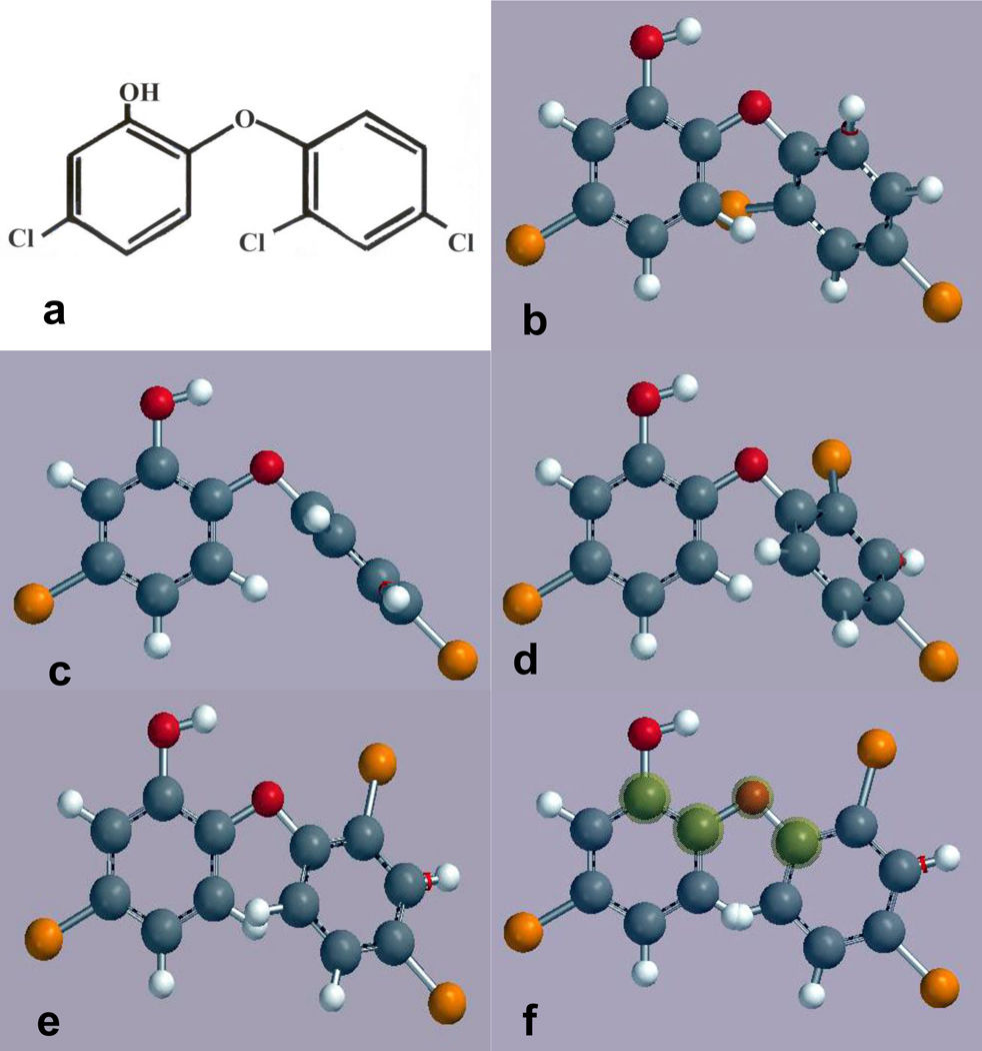
Triclosan oxygen ether bond rotations from 2D 0.0° rotation through to 180° in 3D: (a) Common 2D structure 0.0° ether bond rotation. (b) 3D structure 45.0° ether bond rotation. (c) 3D structure 90.0° ether bond rotation. (d) 3D structure 120.0° ether bond rotation. (e) 3D structure 150.0° ether bond rotation. (f) 3D structure 180.0° ether bond rotation with steric interaction between opposing aromatic hydrogen atoms.

In order to better understand or appreciate computational chemistry data for triclosan ether oxygen bond rotation conformations to produce a skewed energy minimum at 30°, polymer chain entanglement structure and also alternating vibration bond rotation disruptions of polymer chains a range of rotation angles is shown from 0.0° to 180°, Figure 3. Because of steric interference between the two opposing aromatic chlorine and hydrogen atoms the Spartan software was unable to calculate the 3D structure for the 2D triclosan molecule seen in Figure 3(a). However, as the ether oxygen atom bonds rotate the inner chlorine atom away from the hydrogen atom of the opposite aromatic ring Figure 3(a) through 180 degrees the triclosan molecule is presented for 3D viewing at 45°, Figure 3(b); then 90°, Figure 3(c); 120°, Figure 3(d); 150°, Figure 3(e); and finally a complete 180° rotation, Figure 3(f) similar to the conformation in Figure 1. Notice in Figure 3(f) that steric hindrance between two hydrogen atoms from each phenyl ring interferes with rotation to such an extent that repulsive forces appear to greatly contribute to the screwed energy minimum calculated for 30° in Figure 2(a) and 2(b). Further, in all 3D models seen in Figures 2 and 3 the hydrogen atom of the hydroxyl group rotates with the oxygen atom bond inward toward the ether oxygen atom lone-pair electrons.

## 3. Polymer material properties

Mechanomolecular Theory is advanced with computational conformational analysis of the triclosan molecule with preliminary flexural bend testing demonstrating for Polymer Science regarding how molecules can entangle by bond rotations for increased strength and toughness properties [8]. Further, molecular polarity relationships are considered by similar electron distributions for comparable molecular structures [5,6] to predict better compatibilization between polymer blends and also additives for increasing strength and toughness [8,9]. In addition to triclosan and polymer bond entanglements, with subsequent polymer compatibilization less likelihood of additive leaching release for longer retention particularly is expected for triclosan antimicrobial [8]. Triclosan conformational bond energy computational profile demonstrated proof for the possibility of fluctuating bond rotations that can produce complex interactions between molecules to provide bond entanglement strength for cured polymer toughness in the solid state [8]. Further, polymer strengthening by bond rotation entanglements must be increased more as polymer chains draw together during cure so that triclosan would appear to also provide increased strength with more retention in the polymer through secondary hydrogen bonding and aromatic pi-pi ring stacking by van der Waals forces of attraction [8].

**Figure 4:**
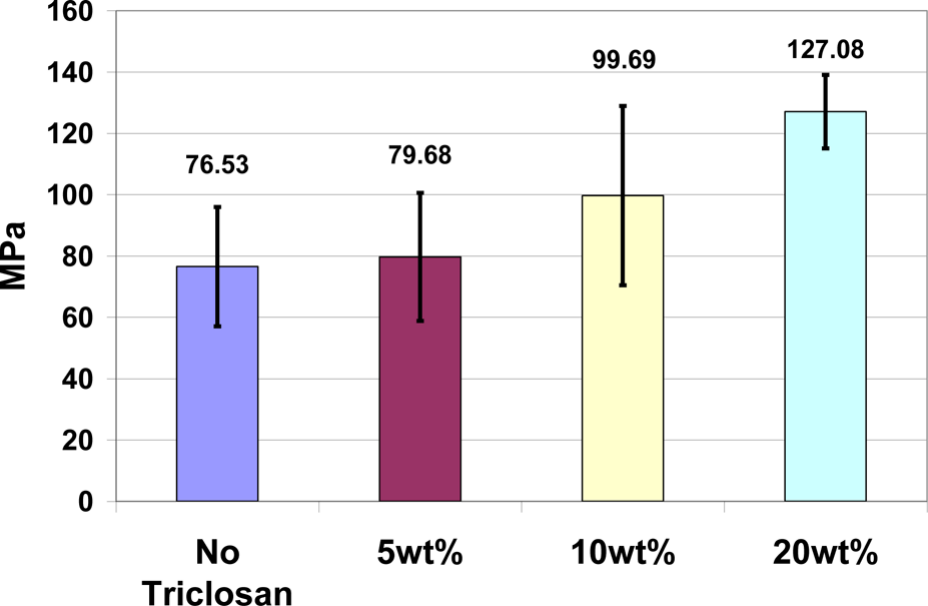
Increasing average flexural strength values for triclosan incorporated into a BisGMA/TEGDMA photocure resin at 0 wt%, 5 wt%, 10 wt% and 20 wt%.

For an example of compatibilization bond rotation entanglements and secondary bonding, incorporation of triclosan to a vinyl ester dimethacrylate copolymer as bisphenol A glycidyl methacrylate (BisGMA) resin and triethyleneglycol dimethacrylate (TEGDMA) diluent at a 2 to 1 ratio with photocuring increased average flexural strengths by additions of 5 wt%, 10 wt% and 20 wt%, Figure 4 [8]. Flexural strength increases between photocure polymers with triclosan and without triclosan were not statistically significantly different for additions of triclosan at 5 wt% and 10 wt% but were significant at 20 wt%, *p* < 0.01. But, for correlation, incorporation of triclosan to the photocure polymer could explain almost 97% of the variability for flexural strength from the average of just 4 values in Figure 4, R^2^ = 0.9656, *p* < 0.05. Increasing triclosan concentrations with visible observation considerably increased flexural bending for all polymer samples tested for a lower modulus. Since strengths increased on average by load-cell testing with clear increased bending for all samples, increasing strain energy and toughness could be interpreted without a stress-strain relationship to integrate force through a distance [8].

**Figure 5:**
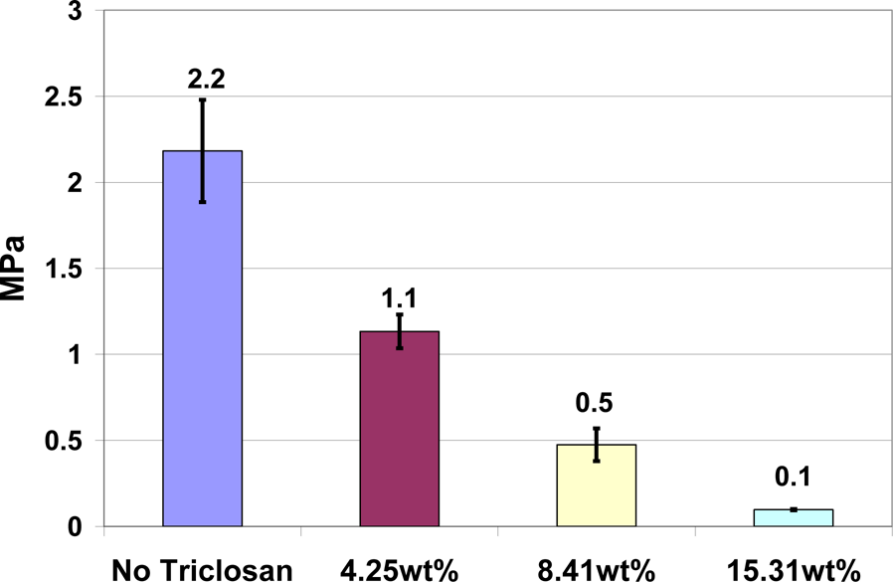
Condensing Index demonstrates increasing loss of paste consistency during compressive force gauge measurements at 0.0 wt%, 4.25 wt%, 8.41 wt% and 15.31 wt% triclosan added into the particulate-filled composite.

Alternatively, triclosan conformational bond rotation computational energy profile provides analysis for the possibility of fluctuating bond rotations that can disrupt weak secondary bonds of attraction to lower viscosity in the resin state [8]. Condensing Index by uniform compressive force gauge measurements, Figure 5, measured lower paste consistency by increasing addition of triclosan to a 2:1 BisGMA-resin:TEGDMA-diluent, 84.5 wt% particulate-filled zirconia silicate photocure composite [8]. Differences between the uncured paste state without triclosan and with 4.25 wt% triclosan were highly significant, *p* < 0.000001. Statistical differences were immensely significant at 8.41 wt% triclosan incorporated into the particulate-filled composite, *p* < 0.00000001. Statistical differences were enormously significant again at 15.31 wt% triclosan incorporated into the particulate-filled composite, *p* < 0.00000001. Correlation for the Condensing Index with loss of composite consistency by addition of triclosan was exponential rather than linear. Although linear correlation was extremely statistically significant, *p* < 10^−13^ and R^2^ = 0.862, exponential correlation explained even more of the variability for loss of composite consistency by incorporation of triclosan, R^2^ = 0.9662 and a *p*-value was not available.

Therefore, in addition to the well-known property as a polymer antimicrobial, new properties with nonpolar triclosan include polymer additive applications as both a nonpolar toughening agent and also a hydrophobic wetting agent [8].

## 4. Antibacterial properties

**Figure 6:**
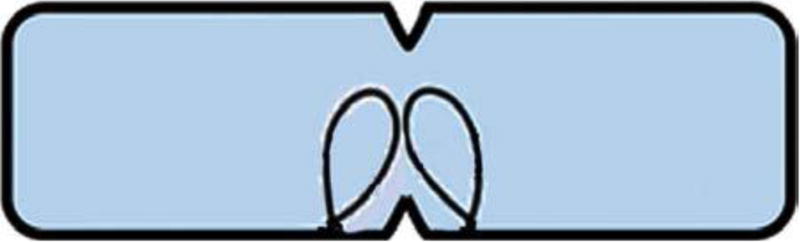
Bacteria with no nucleus attach chromosomes to the cell membrane that subsequently invaginates inward between the two circular-like chromosomes with a septum during the rapid binary fission process.

Triclosan has broad spectrum antibacterial properties influenced by competing actions that break down lipid membranes with rapid fluctuating bond rotations while conversely overly structuring defects into cell membranes that require pliability [8]. The rapid triclosan fluctuations disrupt the phospholipids of the cell membrane so that generalized cell membrane disturbances by triclosan have been shown without cell leakage [10]. Membrane disruptions by triclosan include increasing molecular lipid movement with the breaking of van der Waals forces of attraction between lipid chains, increased lipid chain motion with lower lipid viscosity, reduced lipid membrane density and lower lipid gel to liquid transition temperatures [10,11]. Also, triclosan decreases the melting temperature of mammalian membrane lipids [12]. Triclosan is particularly effective as a result of hydrophobic or nonpolar molecular properties that concentrate triclosan toward the cell membrane phosphate head groups [10,11]. The triclosan accumulating effect toward the bacterial phospholipid membrane seems to be a chief mode of action of membrane destabilization [10,11] that could be critical during bacterial cell division at the septal area where membrane phospholipids start to concentrate [8]. On the other hand bacteria are less susceptible to triclosan during the stationary phase with no cell division [13]. As triclosan concentrations increase toward bactericidal levels, leakage of the bacterial membrane occurs with loss of intracellular components causing disruptions in cell metabolism and hydrolytic enzymes leading to cell death [14,15]. Because bacteria lack a nucleus that protects the chromosomes, during cell division prokaryote chromosomes are pulled apart rapidly by attachments to the cell membrane, Figure 6 [8]. Consequently, bacterial cell division potentially can be disrupted by triclosan at different nonspecific cell membrane levels especially without the extensive fibrous intracellular cytoskeletal strength that supports mammalian cells [8].

**Figure 7:**
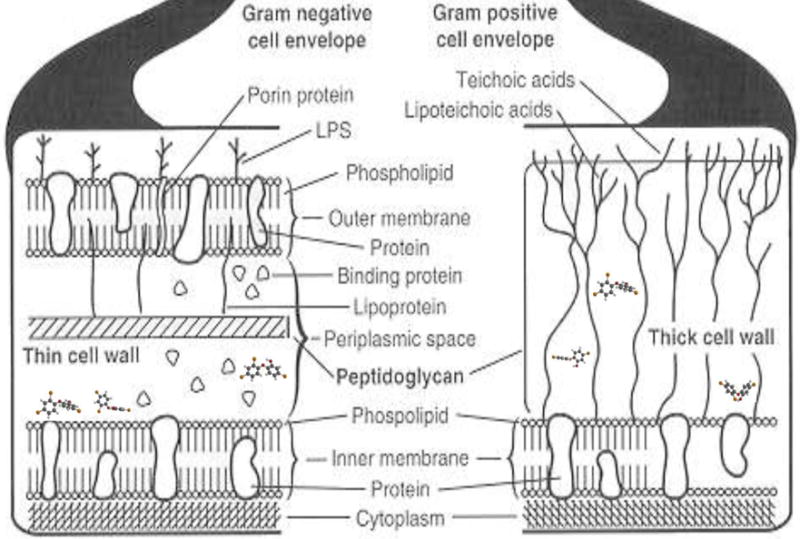
Triclosan molecules approach the bacterial membranes through the cell walls. Resultant vibrational molecular alternating bond rotation fluctuations by triclosan disrupt the membranes. Alternatively, triclosan creates structural defects by bond rotation entanglements or through aromatic intercalating ring stacking by secondary bonding into the phospholipid membranes [8].

During a particularly vulnerable stage of bacterial cell division membrane flexibility is considered an important property when the membrane septum invaginates and phospholipids are forced closer together [8]. Molecular structuring of triclosan into the lipids of the cell membrane particularly during cell division septal invagination is possible with conformational bond rotation entanglements, secondary hydrogen bonding and aromatic pi-pi ring stacking by van der Waals forces of attraction [8]. Further, triclosan has been shown to increase lipid crystallinity with membrane packing not related to entanglements [10,11]. At extremely low triclosan concentrations, a protein that is an enzyme associated with membrane lipids was inhibited through pi-pi ring stacking by van der Waals forces between the aromatic triclosan phenol ring and an aromatic enzyme cofactor nicotinamide adenine dinucleotide (NADH) that also consists of two phosphate groups [16–18]. Computational electron mapping shows that aromatic ring stacking occurs with analogous comparisons between the similar molecular structures of triclosan and NADH [16,17]. Related to membrane structuring, in the bacterial stationary phase with no cell division or septal invagination, aromatic pi-pi ring stacking between triclosan and NADH enzyme inhibition becomes a major factor at the extremely much lower triclosan concentrations [13]. To better illustrate antimicrobial properties, triclosan is shown at a molecular position close to the membranes for both gram negative and gram positive bacteria, Figure 7.

To provide a greater understanding for Molecular Biology and Cell Physiology from Microbiology, as previously indicated conformational bond rotations involving oxygen atom lone-pair electrons by a molecule at an erratic nonpolar-hydrocarbon-membrane/polar-biologic-fluid interface exist as rapid irregular fluctuations. Subsequent triclosan alternating bond rotations then might become sufficiently unstable to provide free potential energy for mechanomolecular disruptions of weaker microbial membranes [8]. In terms of cell physiologic molecular agitations, uneven bond rotations or rapidly irregular nitrogen inversions by multiple other biologic molecules at a highly variable membrane/biologic-fluid interface may also deliver vibration energy for free membrane transport of molecules into cells [8]. Further, molecular conformational bond rotations or inversions may present efficient energetic fluctuations by attached molecules on the outer surface of a plasma membrane for cell signaling, allow unique molecular movements for cell recognition and supply an array of actions by molecules for cell defense [8]. Further, mechanomolecular energy movements in soluble proteins could also generate enzyme mixing to speed reactions [8].

## 5. Triclosan incorporation in polymers

Triclosan has been incorporated into different polymers with wide degrees of success. Triclosan is used in plastic to inhibit material degradation, reduce odors, and lessen discoloration [3]. However, triclosan is used as a plastic additive to a limited degree when compared to the major use in cosmetic and personal care products or for therapeutic purposes [3]. Concentrations of triclosan for plastics have been reported to range from >0.6% to ≤10% [3]. Applications for general use of triclosan in plastics include chopping boards, kitchen utensils, sponges, appliances, gloves, kitchen and bathroom fixtures, medical devices, children’s toys, high chairs, carpets, food storage containers, cling wrap, toilet seats, swimming pool liners, toothbrushes, pet accessories, and flooring materials [1–3]. Consequently widespread utilization of triclosan in non-healthcare applications has been questioned with regard to concerns toward the development of bacterial resistance in therapeutic uses discussed later.

Triclosan was incorporated at 1.0 wt% into a free-radical cure thermoset crosslinked polymer with a vinyl ester dimethacrylate copolymer BisGMA and TEGDMA as a particulate-filled dental composite intended for fillings in 1995 [19]. Triclosan is nonpolar and just soluble in water with only 10 μg/mL at 20 °C (10^−5^ g/mL or 0.001 grams/100 grams water) [1–3]. However, as a result of strong compatible conformation bond rotation entanglements and secondary bonding with the polymer matrix [8], the nonpolar triclosan eluted in aqueous solution at just the smallest amount with 0.02 μg/mL after 24 hours and only 0.08 μg/mL after 56 days [19]. Nevertheless, with a triclosan minimum inhibitory concentration (MIC) of 5 μg/mL *Streptococcus mutans* was inhibited in a similar size broth solution and further was inhibited for substrate polymer adherence shown by scanning electron microscopy (SEM) [19]. Consequently, triclosan was thought to reduce bacterial growth and polymer adherence directly from the polymer surface with minimum antimicrobial release [19]. As an explanation, bacterial inhibition was considered by triclosan molecular mechanical agitation on weak bacterial membranes [8]. Further, bacterial inhibition includes possible membrane structural bond rotation entanglements with secondary bonding defects [8]. Both forms of bacterial inhibition can be disruptive particularly during the growth log phase when actively dividing cells require correct membrane fluidity [8]. Also, secondary bonding between bacteria and the polymer was considered to be interrupted by triclosan vibrational fluctuating mechanomolecular bond rotations as a possible mechanism to prevent microbial surface attachments [8,19]. In another study triclosan demonstrated bacterial inhibition at concentrations 100 times lower than the MICs with actively dividing cells such that triclosan binding to the bacterial cells was considered as a means to enhance membrane fluidity and membrane transport [15]. As a result of the triclosan effectiveness at extremely low elution concentrations for release from the polymer, long-term polymer retention by triclosan appeared to be an excellent antimicrobial benefit [19] that also increased polymer strength and toughness [8]. However, as a practical problem triclosan incorporation into a dental filling composite appears difficult for application due to triclosan molecular bond rotation fluctuations that disrupt secondary bonds between resin monomer chains and also connecting nanoparticulate needed to produce a thickened paste consistency for packing during material insertion into a prepared cavity [8]. Consequently, triclosan creates a gluey sticky particulate-filled dental composite that is impossible to insert as a cavity filling material without producing excessive voids [8].

Later triclosan was incorporated for free-radical curing into a commercial primer for dental resin composites [20] and commercial temporary dental cement [21]. However, with lower resin viscosity triclosan use in a dental primer and cement will not interfere with placement due to the expected secondary bonding disruptions that reduce consistency. In fact, triclosan will help with such bonding application before cure with improved flowability into surface pore nanospaces [8]. Dental cement as BisGMA and TEGDMA with 40 wt% silica and alumina was evaluated for triclosan distribution uniformity at 3 wt% and 1 wt% by heating cured samples in an electric stove up to 160 °C and maintained for 60 minutes for full removal of the particulate antibacterial agent with subsequent characterization of empty spaces by SEM. Also, evidence of triclosan chlorine groups was identified by electron dispersive spectroscopy (EDX) microprobe coupled to the SEM before heating [22]. Triclosan white crystalline particulate has a melting point of 54–57 °C and decomposition temperature greater than 280 °C so that full elimination from the dental cement was accomplished by the heating method above without loss of the resin component all of which has demonstrated uniform distribution of the triclosan antimicrobial particulate [22].

Recently, triclosan has been added at 10 wt% into a free-radical photocure discontinuous fiber-reinforced dental composite with nonstatistical stronger mechanical test results that include excellent compound molding viscosity consistency for placement into a filling cavity [8]. Further, triclosan was incorporated at 10 wt% into a free-radical chemical cure acrylic bone cement model to produce a stronger material [8]. Also, the triclosan nonpolar molecular property was considered as an important characteristic that could interfere by separating away from polar or hydrophilic bonding techniques common in dentistry [8].

Thermoplastic polymers that soften when heated above the glass transition temperature (Tg) and where molecular chains start to move but then harden on cooling have been used with a melt mixing processing technique to incorporate triclosan [23–25]. Polypropylene was tested for triclosan incorporation with commercial products showing antibacterial properties against common bacteria and also found not to leach readily from the polymer for long-term retention [23]. Further, following SEM imaging triclosan was found to move through polypropylene following surface cleaning and aggregate at the surface into particles over 1 um long after an hour [personal communication Swofford, HW (2003) Director of R&D, Migration of Microban® Active. Scanning Electron Microscopy Imaging of Triclosan Microban “B” Migration in Polyethylene Film 10,000× Magnification 0.5% Triclosan Cleaned Surface through 60 minutes. Microban Products Company, Huntersville, NC]. Polystyrene incorporated with triclosan was able to inhibit bacteria over short periods and similarly did not diffuse readily out of the polymer [24]. Overall, triclosan incorporation into a polymer was considered as an effective method toward improving current hygienic practices [23]. However, for more-lasting use with limited bioavailability release from a polymer acrylonitrile-butadiene-styrene (ABS) plastic made commercially available with triclosan at 5 wt% was not found to inhibit thickened biofilm colonization over longer extended periods of 1 to 3 weeks [25].

Cyanoacrylate adhesives that polymerize at room temperature when exposed to atmospheric moisture [26] have been used as sutures to glue tissues together [26–28]. The bond strength of a cyanoacrylate wound closure compared to a suture treated wound closure is about one half [27,29]. An effort to improve cyanoacrylate bond strength with nanosilica or carbon nanotubes was investigated with the incorporation of 5% triclosan that is expected to release slowly and subsequently demonstrated antimicrobial properties up to 5 days in an animal [28]. Also, addition of triclosan to the cyanoacrylate adhesive reduced white blood cell count better than wounds treated by bandage that indicates a better antimicrobial effect [28].

## 6. Triclosan coatings

Triclosan has been applied by many different coating processes. Several proprietary methods have been completed commercially to include coating absorbable polymer sutures and also polyester or Dacron vascular grafts. Synthetic polymer absorbable sutures have rapidly emerged as one of the fastest growing suture markets [30]. The most common absorbable sutures are based on cyclic monomers that open the ring molecular structure during polymerization. High crystallinity of the absorbable polymers or copolymers determines suture strength whereas low crystallinity decreases the time for suture degradation [30]. Surgical site infections (SSIs) commonly account for a majority of post-surgical complications [31–34]. In order to reduce SSIs following conventional surgery, proprietary coatings of absorbable sutures were developed with triclosan [31–34]. The three most common polymers used with a triclosan coating include polyglactin, poliglecaprone and polydioxanone [31–34]. As a result, use of Triclosan coated absorbable sutures has been shown to reduce the incidence of SSIs compared to non-antibacterial coated sutures in multiple published studies [31–35]. Of serious consequence, vascular graft infections pose threats with complications often leading to a relatively high mortality rate where use of triclosan in coatings for experimental polyester and Dacron models indicate a positive antibacterial property for treatment [36,37].

Triclosan is highly soluble in organic solvents [1,3] so that coatings are achievable through a dip and evaporation process [38,39]. Urinary catheters have been coated by dissolving triclosan and other antimicrobial combinations in chloroform with evaporation of the solvent to prevent bacterial colonization of some common uropathogens for 7–12 weeks in vitro [38]. A unique property for the triclosan molecule to provide mechanomolecular energy through rapid conformational ether bond rotation fluctuations [8] is probably associated with demonstrated ability for the antimicrobial to diffuse through silicone balloons and elute into all-silicone catheters for inhibition of bacterial colonization [40]. Further, triclosan has been grafted to a polyethylene polymer surface after a cold plasma coating process with acrylic acid that overcomes problems with the hydrophobic or nonpolar nature of polyethylene [41]. After triclosan was grafted to the polyethylene acrylic acid treated surface microbial adherence was then inhibited [41].

## 7. Triclosan in toothpaste copolymer

Triclosan does not bind to the gingival tissue effectively and so does not deliver a continued level of anti-plaque protection [42–44]. To increase uptake and retention of triclosan by oral gingival and tooth surfaces for the improvement of plaque control, 2% polyvinyl methyl ether/maleic acid copolymer (PVM/MA) is used as a delivery system for 0.3% triclosan in toothpaste [42–44]. PVM/MA also increases retention of triclosan in both plaque and saliva [42]. The PVM/MA copolymer has a PVM compatible component to make triclosan soluble for retention in micelle surfactant groups and an MA carboxyl attachment group in the liquid coat that can react with oral surfaces by way of calcium so that triclosan is released slowly by the salivary aqueous medium [45]. Triclosan with PVM/MA has been shown to produce both antiplaque and antigingivitis effects [43–47] and further reduce the severity of calculus [42,45,48]. Also, an anticaries effect has been observed with triclosan [45].

## 8. Bacterial resistance to triclosan not seen under normal conditions

Triclosan is the most studied biocide in terms of bacterial resistance [2]. In a non-clinical test a specific bacterial resistance mechanism under laboratory conditions at low triclosan picomolar concentrations has been associated with cell membrane lipids through inhibiting a protein that is an enzyme as a target [16–18]. But, clinical relevance in multiple studies for bacterial resistance has not been demonstrated for about 50 years of long-term repeated use of triclosan [2,3,49–52]. In one individual long-term clinical study there was no alteration in oral antimicrobial resistance from patients using 0.3% triclosan with the copolymer PVM/MA for at least 5 years [53,54]. Several government reports from large groups of authorities in the field have determined that although cross-resistance to antimicrobials or antibiotics can be demonstrated under artificial laboratory conditions, the development of resistance in natural or clinical environments with complex multiple bacterial species was not an equivalent condition for the use of triclosan [2,3,49]. Further, one governmental report advocated use of triclosan where a Health benefit is found [2]. The chief concern for bacterial resistance was for widespread application in non-health related uses, for example in cosmetics, clothing, food contact plastics, toys, carpets, food storage containers, and other non-health purposes [2]. Regardless, in cosmetic or over-the-counter products triclosan was considered safe and did not create a threat to form antimicrobial resistant bacteria [2,3,49]. Also, conclusions from the government documents determined that triclosan had a low potential for acquired bacterial resistance [2,3,50]. Triclosan has even been shown to provide better health defense against bacteria than standard antibiotics in an acute animal infection model with tetracycline and ampicillin [55]. Triclosan has been an important antimicrobial clinically to destroy methicillin-resistant Staph Aureus (MRSA) and used as surgical scrubs, hand washing and body wash for MRSA carriers prior to surgery [2]. Also, triclosan has shown synergism with antibiotic therapy clinically for seven bacterial species [2].

## 9. Toxicology profile

Triclosan has shown efficacy and safety throughout long term application [1–3,49,50,52,56]. Triclosan is rapidly adsorbed 100 percent from the gastrointestinal tract and can also adsorb dermally [2,3]. Triclosan is removed rapidly from the blood and excreted primarily in the urine [3]. Triclosan has not shown any evidence of bioaccumulation [3]. Triclosan has low acute oral and dermal toxicity [3]. Further, triclosan has not shown any evidence of carcinogenic potential [3]. In 2010 without sufficient supporting evidence the Food and Drug Administration (FDA) released a statement with recommendation that the consumer use of products containing triclosan not be changed [57]. The FDA approved use of Colgate’s Total with triclosan at 0.3 wt% in 1997 with expected full solubility release after 24 hours of over 10 ug/mL at 37 °C. However, release of triclosan from a plastic polymer is extremely small [19,23,24]. For example, triclosan incorporated at 1.0 wt% eluted from a BisGMA/TEGDMA dental composite in solution at just negligible amounts with 0.02 μg/mL after 24 hours and only 0.08 μg/mL after 56 days [19]. Further, triclosan in a dental polymer sealing material Seal and Protect has been sold commercially since at least 2005 [20] and at a reported concentration of 2.5–10 wt% from 2013 [58]. So, in terms of solubility release, triclosan incorporation into a polymer plastic or composite should be easy to accommodate guidelines that would not contradict the FDA recommendation for current use of triclosan.

## 10. Conclusions

Triclosan computational chemistry conformational bond rotation energy profile with dipoles near the ether oxygen atom lone pair electrons help to better explain mechanomolecular movements that can fluctuate rapidly between polar and nonpolar mediums. Therefore, the interface between a cell membrane and biologic fluid should create microenvironmental conditions for triclosan to produce an exaggerated form of free mechanomolecular energy. Consequently, weaker bacterial membranes are susceptible to disruption by triclosan. Alternatively, triclosan can structure a cell membrane by bond rotation entanglements or also secondary bonding to create a defect that reduces membrane pliability needed by bacteria during cell division. Triclosan as a white particulate powder can be incorporated easily into a polymer as an antimicrobial additive. Further, the triclosan additive by bond entanglements and secondary bonding increases polymer toughness and strength as a nonpolar toughening agent. Thus, nonpolar triclosan polymer compatibilization with bond entanglements and secondary bonding provides long-term antimicrobial retention with minimum release into aqueous media. Also, triclosan supplies vibrational energy to break up secondary bonding in the resin state to reduce viscosity and so ease mixing and blending requirements as a hydrophobic wetting agent. Triclosan has been added into a polymer system by mixing incorporation before cure into the resin state, by melt mixing with thermoplastics and through different coatings that can be particularly easy as triclosan is soluble in organic solvents for subsequent application by a dip method and then dried. Clinical significance for bacterial resistance to triclosan has not been shown for almost 50 years of long-term frequent use of triclosan so that a government report has further advocated the use of triclosan where a health benefit is found.

## Supplementary Material

Spartan Calculations for Bond Rotations
