# Supplementary material for: Triclosan antimicrobial polymers
Source: AIMS Mol Sci. Author manuscript; Available in PMC 2016 Jun 6. (PMC4893770; doi:10.3934/molsci.2016.1.88)
Supplement: Spartan Calculations for Bond Rotations [file NIHMS788831-supplement-Spartan_Calculations_for_Bond_Rotations.doc]

**SPARTAN CALCULATIONS FOR BOND ROTATION AND ENERGIES**

**Dr. Richard C. Petersen***

**University of Alabama at Birmingham**

*corresponding author [richbme@uab.edu](mailto:richbme@uab.edu)

**Abstract**

Spartan software calculations were made available through Sean W. Ohlingher, Vice President and Technical Support, Wavefunction Inc, Newport Beach, CA and presented with the following results.


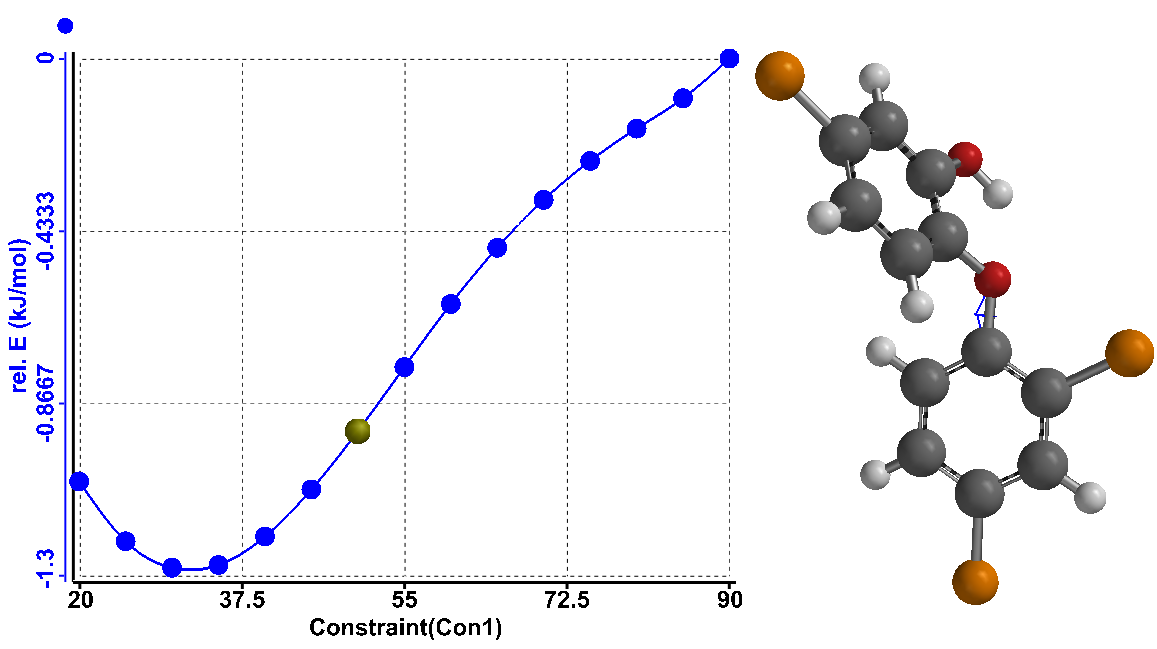


Figure 1. Bond Rotations and Energies for Triclosan with Molecular Structure at approximately 50 Degree Oxygen Ether Bond Rotation

Spartan '04

build 122 (Jan 8 2004)

Wavefunction Developers:

B.J. Deppmeier, A.J. Driessen, T.S. Hehre, W.J. Hehre,

J.A. Johnson, P.E. Klunzinger, J.M. Leonard, I.N. Pham

W.J. Pietro, Jianguo Yu

Q-Chem Developers:

J. Kong, C.A. White, A.I. Krylov, C.D. Sherrill,

R.D. Adamson, T.R. Furlani, M.S. Lee, A.M. Lee,

S.R. Gwaltney, T.R. Adams, C. Ochsenfeld, A.T.B. Gilbert,

G.S. Kedziora, V.A. Rassolov, D. R. Maurice, N. Nair,

Y. Shao, N.A. Besley, P.E. Maslen, J.P. Dombroski,

H. Dachsel, W.M. Zhang, P.P. Korambath, J. Baker,

E.F. C. Byrd, T. Van Voorhis, M. Oumi, S. Hirata,

C.P. Hsu, N. Ishikawa, J. Florian, A. Warshel,

B.G. Johnson, P.M.W. Gill, M. Head-Gordon, J.A. Pople

Wavefunction Inc. Sales: sales@wavefun.com

Irvine CA Support: support@wavefun.com

Web: www.wavefun.com

Copyright © 1995 - 2003

----------------------------------------------------

Spartan 'O4 Quantum Mechanics Module 122

Windows PC (Intel x86)

**Table 1 Calculations for Bond Rotational and Energy Chart**

--------------------------------------------------------------

User input:

--------------------------------------------------------------

$comment

M001

$end

$molecule

0 1

6 -2.2314548462 -0.94161591324 0.85158935862

6 -2.7989944799 0.90070391643 -1.1283315197

6 -1.2474245498 -0.56898449851 -0.043823528661

6 -3.4996075766 -0.40357292309 0.75743855721

6 -3.7743334675 0.51682817838 -0.23028449511

6 -1.5361331491 0.35313304148 -1.0314125405

1 -4.2569466486 -0.69965703266 1.4515088566

1 -0.77044916752 0.6362034186 -1.7235984935

1 -3.0254862576 1.6126147834 -1.8938179594

8 -0.0037744078697 -1.1638509912 0.050557517656

6 1.1866564481 -0.42599937538 0.039283380197

6 3.627365583 0.7981614787 -0.044477503192

6 1.3263986883 0.83253126259 0.56846521644

6 2.2593213761 -1.0919033804 -0.52811958831

6 3.4892688712 -0.47134573632 -0.56690928119

6 2.5603729235 1.459838329 0.52522063587

1 0.48709163648 1.3265695588 1.0117056195

1 4.3156871623 -0.98650827162 -1.0052028003

17 -1.879866737 -2.0961699524 2.0959707173

17 -5.3741365052 1.2000406653 -0.34296247121

1 2.6878252695 2.4406061529 0.9313231702

17 5.1891341456 1.57616081 -0.10495757399

8 2.1142737697 -2.3469106064 -1.0421936294

1 1.2205959163 -2.6828440196 -0.88278404795

$end

$rem

JOBTYPE OPT

TIDY_SYM TRUE

EXCHANGE HF

BASIS 3-21G*

SYMMETRY FALSE

USE_SP_DERIV 2

GEOM_OPT_HESSIAN READ

EXTERNAL_HESSIAN TRUE

GUI GUI_SPARTAN

TERSE_OUTPUT TRUE

$end

$opt

CONSTRAINT

tors 6 3 10 11 50.000000

ENDCONSTRAINT

$end

--------------------------------------------------------------

Processing $rem in C:\DOCUMENTS AND SETTINGS\ALL USERS\DOCUMENTS\SPARTAN'04 DEVELOPMENT VERSION\auxdir\preferences.

(Site specific preferences.)

... THRESH 9

... SCF_CONVERGENCE 7

... SMALL_PROD_XCMAT 9

... BASIS_LIN_DEP_THRESH 5

... GUI GUI_SPARTAN

... TERSE_OUTPUT TRUE

Processing $rem in system registry

... MEM_TOTAL 500 # MB

Processing $rem in the input.

... JOBTYPE OPT

... TIDY_SYM TRUE

... EXCHANGE HF

... BASIS 3-21G*

... SYMMETRY FALSE

... USE_SP_DERIV 2

... GEOM_OPT_HESSIAN READ

... EXTERNAL_HESSIAN TRUE

... GUI GUI_SPARTAN

... TERSE_OUTPUT TRUE

Total Memory Limit in MB = 500

Mega-Array Size in MB = 31

#####################################################

# Entering fldman.exe on Tue May 25 14:56:50 2004 #

#####################################################

Requested basis set is 3-21G(d)

There are 71 shells and 197 basis functions

#####################################################

# Entering gesman.exe on Tue May 25 14:56:50 2004 #

#####################################################

Smallest overlap matrix eigenvalue = 1.33E-003

Multipole matrices computed through 2nd order

#####################################################

# Entering scfman.exe on Tue May 25 14:56:51 2004 #

#####################################################

A restricted Hartree-Fock SCF calculation will be

performed using Pulay DIIS extrapolation

SCF converges when DIIS error is below 1.0E-007

---------------------------------------

Cycle Energy DIIS Error

---------------------------------------

1 -1984.0469582445 8.31E-002

2 -1976.8163925110 5.67E-003

3 -1976.9900842447 2.68E-003

4 -1977.0231504227 6.84E-004

5 -1977.0266392173 1.81E-004

6 -1977.0268626454 6.35E-005

7 -1977.0269055543 2.26E-005

8 -1977.0269116314 8.14E-006

9 -1977.0269126142 3.22E-006

10 -1977.0269127874 1.44E-006

11 -1977.0269128320 6.56E-007

12 -1977.0269128455 2.10E-007

13 -1977.0269128337 5.71E-008 Convergence criterion met

---------------------------------------

SCF time: CPU 43.92 s wall 46265.00 s

#####################################################

# Entering anlman.exe on Tue May 25 14:57:38 2004 #

#####################################################

+++F6+++

Analysis of SCF Wavefunction

Mulliken Net Atomic Charges

Atom Charge (a.u.)

----------------------------------------

1 C -0.234390

2 C -0.206471

3 C 0.400745

4 C -0.173866

5 C -0.207346

6 C -0.235720

7 H 0.306545

8 H 0.288596

9 H 0.288479

10 O -0.790853

11 C 0.309480

12 C -0.202511

13 C -0.231221

14 C 0.378992

15 C -0.207248

16 C -0.222446

17 H 0.281755

18 H 0.299870

19 Cl 0.095807

20 Cl 0.062886

21 H 0.278137

22 Cl 0.050231

23 O -0.751666

24 H 0.422214

----------------------------------------

Sum of atomic charges = 0.000000

-----------------------------------------------------------------

Cartesian Multipole Moments

-----------------------------------------------------------------

Charge (ESU x 10^10)

0.0000

Dipole Moment (Debye)

X -1.9640 Y 1.4841 Z -0.5499

Tot 2.5224

Quadrupole Moments (Debye-Ang)

XX -133.2599 XY 0.6529 YY -120.4761

XZ 0.0215 YZ -0.3011 ZZ -119.8914

Traceless Quadrupole Moments (Debye-Ang)

QXX -26.1523 QYY 12.1990 QZZ 13.9533

QXY 1.9588 QXZ 0.0644 QYZ -0.9032

Octapole Moments (Debye-Ang^2)

XXX 37.2661 XXY -12.3120 XYY 13.8247

YYY 16.1525 XXZ -13.1999 YZ 7.3463

YYZ -17.6687 XZZ 9.4599 YZZ 11.8387

ZZZ -64.8871

Traceless Octapole Moments (Debye-Ang^2)

XXX 14.0351 YYY 101.1754 ZZZ -111.5058

XXY -231.7178 XXZ 89.2689 XYY 25.7177

XYZ 110.1947 XZZ -39.7528 YYZ 22.2369

YZZ 130.5424

Hexadecapole Moments (Debye-Ang^3)

XXXX -8016.3740 XXXY -183.4359 XXYY -1567.1526

XYYY -148.3167 YYYY -1301.0619 XXXZ -103.9537

XXYZ 17.4499 XYYZ 42.1754 YYYZ 129.7738

XXZZ -1424.2049 XYZZ -56.4987 YYZZ -315.7714

XZZZ 13.1097 YZZZ 112.5100 ZZZZ -675.4715

Traceless Hexadecapole Moments (Debye-Ang^3)

XXXX -487.9260 XXXY -1789.4640 XXXZ -8725.0490

XXYY -1496.7571 XXYZ -2056.1168 XXZZ 1984.6831

XYYY 1898.0540 XYYZ 5158.4495 XYZZ -108.5900

XZZZ 3566.5994 YYYY 482.7402 YYYZ 1961.1849

YYZZ 1014.0170 YZZZ 94.9319 ZZZZ -2998.7001

-----------------------------------------------------------------

---F6---

#####################################################

# Entering drvman.exe on Tue May 25 14:57:38 2004 #

#####################################################

Calculating analytic gradient of the SCF energy

Spartan '04 Fast HF Program: (PC/x86) Release 122

Calculation started: Tue May 25 14:57:39 2004

JOBNAME.TEMP

Run type: Molecular gradient (no SCF)

Model: RHF/ABASIS

Number of shells: 71

31 S shells

37 SP shells

3 6D shells

Number of basis functions: 197

Number of electrons: 146

Number of heavy atoms: 17

Number of hydrogens: 7

Use of molecular symmetry disabled

Molecular charge: 0

Spin multiplicity: 1

Memory model: direct 13.0 Mb

Point Group = C1 Order = 1 Nsymop = 1

This system has 0 degrees of freedom

Cartesian gradient (a.u.)

Atom X Y Z

---- ------------ ------------ ------------

C 1 -0.0002008 0.0001526 -0.0000299

C 2 -0.0000637 -0.0000011 -0.0001461

C 3 -0.0005995 0.0002369 0.0008379

C 4 -0.0002287 0.0000172 0.0000251

C 5 0.0001423 -0.0000905 0.0000830

C 6 0.0004582 -0.0001626 0.0003548

H 7 -0.0000568 0.0000058 0.0000162

H 8 0.0002501 -0.0000343 -0.0000166

H 9 -0.0000108 -0.0000093 0.0000518

O 10 0.0004814 -0.0005781 -0.0010994

C 11 0.0002614 0.0003655 0.0002696

C 12 -0.0002019 -0.0000015 0.0000399

C 13 -0.0002112 -0.0000385 -0.0003684

C 14 0.0000392 0.0001868 0.0001395

C 15 -0.0000845 -0.0000923 -0.0000321

C 16 0.0000070 -0.0000546 -0.0000271

H 17 -0.0000311 -0.0000245 -0.0000661

H 18 -0.0000747 0.0000712 0.0000285

Cl 19 -0.0000965 0.0001097 -0.0000789

Cl 20 0.0000740 -0.0000537 0.0000200

H 21 0.0000196 0.0000001 -0.0000248

Cl 22 0.0001629 0.0000628 0.0000389

O 23 0.0000419 -0.0000617 -0.0000568

H 24 -0.0000777 -0.0000057 0.0000411

E(HF) = -1977.0269128 a.u.

Reason for exit: Successful completion

Fast HF Program CPU Time : 000:00:12.3

Fast HF Program Wall Time: 000:00:12.8

Gradient of SCF Energy

1 2 3 4 5 6

1 -0.0002008 -0.0000637 -0.0005995 -0.0002287 0.0001423 0.0004582

2 0.0001526 -0.0000011 0.0002369 0.0000172 -0.0000905 -0.0001626

3 -0.0000299 -0.0001461 0.0008379 0.0000251 0.0000830 0.0003548

7 8 9 10 11 12

1 -0.0000568 0.0002501 -0.0000108 0.0004814 0.0002614 -0.0002019

2 0.0000058 -0.0000343 -0.0000093 -0.0005781 0.0003655 -0.0000015

3 0.0000162 -0.0000166 0.0000518 -0.0010994 0.0002696 0.0000399

13 14 15 16 17 18

1 -0.0002112 0.0000392 -0.0000845 0.0000070 -0.0000311 -0.0000747

2 -0.0000385 0.0001868 -0.0000923 -0.0000546 -0.0000245 0.0000712

3 -0.0003684 0.0001395 -0.0000321 -0.0000271 -0.0000661 0.0000285

19 20 21 22 23 24

1 -0.0000965 0.0000740 0.0000196 0.0001629 0.0000419 -0.0000777

2 0.0001097 -0.0000537 0.0000001 0.0000628 -0.0000617 -0.0000057

3 -0.0000789 0.0000200 -0.0000248 0.0000389 -0.0000568 0.0000411

Max gradient component = 1.099E-003

RMS gradient = 2.410E-004

Gradient time: CPU 13.02 s wall 13578.00 s

+++F6+++

---F6---

#####################################################

# Entering optman.exe on Tue May 25 14:57:52 2004 #

#####################################################

#####################################################

# Entering fldman.exe on Tue May 25 14:57:53 2004 #

#####################################################

Requested basis set is 3-21G(d)

There are 71 shells and 197 basis functions

Applying Cartesian multipole field

Component Value

--------- -----

(2,0,0) 1.00000E-010

(0,2,0) 2.00000E-010

(0,0,2) -3.00000E-010

#####################################################

# Entering gesman.exe on Tue May 25 14:57:53 2004 #

#####################################################

Smallest overlap matrix eigenvalue = 1.33E-003

Multipole matrices computed through 2nd order

#####################################################

# Entering scfman.exe on Tue May 25 14:57:53 2004 #

#####################################################

A restricted Hartree-Fock SCF calculation will be

performed using Pulay DIIS extrapolation

SCF converges when DIIS error is below 1.0E-007

---------------------------------------

Cycle Energy DIIS Error

---------------------------------------

1 -1977.0284510912 1.77E-003

2 -1977.0262013042 2.18E-004

3 -1977.0267930827 7.59E-005

4 -1977.0268843009 1.93E-005

5 -1977.0268879530 8.42E-006

6 -1977.0268887252 2.16E-006

7 -1977.0268887938 7.89E-007

8 -1977.0268888057 3.16E-007

9 -1977.0268887991 1.50E-007

10 -1977.0268887327 6.51E-008 Convergence criterion met

---------------------------------------

SCF time: CPU 32.63 s wall 35282.00 s

#####################################################

# Entering anlman.exe on Tue May 25 14:58:30 2004 #

#####################################################

+++F6+++

Analysis of SCF Wavefunction

Mulliken Net Atomic Charges

Atom Charge (a.u.)

----------------------------------------

1 C -0.234898

2 C -0.206353

3 C 0.399276

4 C -0.173737

5 C -0.207575

6 C -0.234242

7 H 0.306301

8 H 0.287863

9 H 0.288455

10 O -0.788806

11 C 0.304868

12 C -0.202081

13 C -0.228563

14 C 0.382577

15 C -0.207197

16 C -0.223254

17 H 0.281689

18 H 0.299762

19 Cl 0.095837

20 Cl 0.062962

21 H 0.278019

22 Cl 0.050586

23 O -0.753434

24 H 0.421944

----------------------------------------

Sum of atomic charges = 0.000000

-----------------------------------------------------------------

Cartesian Multipole Moments

-----------------------------------------------------------------

Charge (ESU x 10^10)

0.0000

Dipole Moment (Debye)

X -1.9124 Y 1.4233 Z -0.5386

Tot 2.4440

Quadrupole Moments (Debye-Ang)

XX -133.1965 XY 0.6132 YY -120.7334

XZ 0.1074 YZ -0.0687 ZZ -119.5582

Traceless Quadrupole Moments (Debye-Ang)

QXX -26.1014 QYY 11.2880 QZZ 14.8133

QXY 1.8395 QXZ 0.3223 QYZ -0.2062

Octapole Moments (Debye-Ang^2)

XXX 37.6522 XXY -12.5073 XYY 14.5793

YYY 12.7045 XXZ -13.1168 YZ 7.2673

YYZ -17.4904 XZZ 9.5615 YZZ 12.0374

ZZZ -64.5464

Traceless Octapole Moments (Debye-Ang^2)

XXX 8.6463 YYY 80.4557 ZZZ -111.8132

XXY -224.3130 XXZ 88.7086 XYY 33.3101

XYZ 109.0097 XZZ -41.9564 YYZ 23.1047

YZZ 143.8572

Hexadecapole Moments (Debye-Ang^3)

XXXX -8018.3044 XXXY -188.8135 XXYY -1560.3737

XYYY -155.7203 YYYY -1260.7199 XXXZ -112.0228

XXYZ 19.5040 XYYZ 40.9935 YYYZ 132.8325

XXZZ -1427.9447 XYZZ -57.7144 YYZZ -315.4432

XZZZ 10.6519 YZZZ 113.0553 ZZZZ -714.8721

Traceless Hexadecapole Moments (Debye-Ang^3)

XXXX -738.6834 XXXY -1724.2465 XXXZ -9045.4112

XXYY -1496.1043 XXYZ -1932.1242 XXZZ 2234.7877

XYYY 1750.5348 XYYZ 5209.9792 XYZZ -26.2883

XZZZ 3835.4321 YYYY 499.9489 YYYZ 2007.2677

YYZZ 996.1554 YZZZ -75.1436 ZZZZ -3230.9430

-----------------------------------------------------------------

---F6---

#####################################################

# Entering drvman.exe on Tue May 25 14:58:30 2004 #

#####################################################

Calculating analytic gradient of the SCF energy

Spartan '04 Fast HF Program: (PC/x86) Release 122

Calculation started: Tue May 25 14:58:31 2004

JOBNAME.TEMP

Run type: Molecular gradient (no SCF)

Model: RHF/ABASIS

Number of shells: 71

31 S shells

37 SP shells

3 6D shells

Number of basis functions: 197

Number of electrons: 146

Number of heavy atoms: 17

Number of hydrogens: 7

Use of molecular symmetry disabled

Molecular charge: 0

Spin multiplicity: 1

Memory model: direct 13.0 Mb

Point Group = C1 Order = 1 Nsymop = 1

This system has 0 degrees of freedom

Cartesian gradient (a.u.)

Atom X Y Z

---- ------------ ------------ ------------

C 1 0.0000364 -0.0002297 0.0001881

C 2 0.0002284 -0.0000944 0.0001491

C 3 0.0006340 0.0004975 0.0000852

C 4 0.0001336 0.0001727 -0.0003244

C 5 -0.0000278 -0.0000449 0.0000591

C 6 -0.0007157 -0.0001055 -0.0001468

H 7 -0.0000204 -0.0000841 0.0001114

H 8 -0.0000137 0.0001215 -0.0001270

H 9 0.0000179 0.0000624 -0.0000798

O 10 -0.0011597 -0.0005301 0.0000930

C 11 0.0001706 0.0014208 0.0006969

C 12 -0.0004323 -0.0014742 -0.0007397

C 13 -0.0011061 -0.0021457 -0.0008850

C 14 -0.0010497 -0.0006335 -0.0000858

C 15 0.0008551 0.0019628 0.0009634

C 16 0.0009460 0.0000720 -0.0001978

H 17 0.0002270 -0.0007629 -0.0005209

H 18 -0.0001802 0.0007768 0.0004420

Cl 19 0.0000489 0.0000344 -0.0000127

Cl 20 0.0001823 -0.0000493 -0.0000082

H 21 0.0001006 -0.0016265 -0.0009449

Cl 22 0.0011444 0.0002498 -0.0001008

O 23 0.0014116 0.0029360 0.0011862

H 24 -0.0014312 -0.0005260 0.0001994

E(HF) = -1977.0268887 a.u.

Reason for exit: Successful completion

Fast HF Program CPU Time : 000:00:12.5

Fast HF Program Wall Time: 000:00:12.9

Gradient of SCF Energy

1 2 3 4 5 6

1 0.0000364 0.0002284 0.0006340 0.0001336 -0.0000278 -0.0007157

2 -0.0002297 -0.0000944 0.0004975 0.0001727 -0.0000449 -0.0001055

3 0.0001881 0.0001491 0.0000852 -0.0003244 0.0000591 -0.0001468

7 8 9 10 11 12

1 -0.0000204 -0.0000137 0.0000179 -0.0011597 0.0001706 -0.0004323

2 -0.0000841 0.0001215 0.0000624 -0.0005301 0.0014208 -0.0014742

3 0.0001114 -0.0001270 -0.0000798 0.0000930 0.0006969 -0.0007397

13 14 15 16 17 18

1 -0.0011061 -0.0010497 0.0008551 0.0009460 0.0002270 -0.0001802

2 -0.0021457 -0.0006335 0.0019628 0.0000720 -0.0007629 0.0007768

3 -0.0008850 -0.0000858 0.0009634 -0.0001978 -0.0005209 0.0004420

19 20 21 22 23 24

1 0.0000489 0.0001823 0.0001006 0.0011444 0.0014116 -0.0014312

2 0.0000344 -0.0000493 -0.0016265 0.0002498 0.0029360 -0.0005260

3 -0.0000127 -0.0000082 -0.0009449 -0.0001008 0.0011862 0.0001994

Max gradient component = 2.936E-003

RMS gradient = 7.847E-004

Gradient time: CPU 13.20 s wall 13656.00 s

+++F6+++

---F6---

#####################################################

# Entering optman.exe on Tue May 25 14:58:44 2004 #

#####################################################

#####################################################

# Entering fldman.exe on Tue May 25 14:58:45 2004 #

#####################################################

Requested basis set is 3-21G(d)

There are 71 shells and 197 basis functions

Applying Cartesian multipole field

Component Value

--------- -----

(2,0,0) 1.00000E-010

(0,2,0) 2.00000E-010

(0,0,2) -3.00000E-010

#####################################################

# Entering gesman.exe on Tue May 25 14:58:45 2004 #

#####################################################

Smallest overlap matrix eigenvalue = 1.33E-003

Multipole matrices computed through 2nd order

#####################################################

# Entering scfman.exe on Tue May 25 14:58:45 2004 #

#####################################################

A restricted Hartree-Fock SCF calculation will be

performed using Pulay DIIS extrapolation

SCF converges when DIIS error is below 1.0E-007

---------------------------------------

Cycle Energy DIIS Error

---------------------------------------

1 -1977.0214256130 8.68E-004

2 -1977.0267842934 1.05E-004

3 -1977.0269134640 3.78E-005

4 -1977.0269321485 1.87E-005

5 -1977.0269346213 4.39E-006

6 -1977.0269349061 1.58E-006

7 -1977.0269349304 5.29E-007

8 -1977.0269349387 2.01E-007

9 -1977.0269349187 8.46E-008 Convergence criterion met

---------------------------------------

SCF time: CPU 29.86 s wall 36187.00 s

#####################################################

# Entering anlman.exe on Tue May 25 14:59:23 2004 #

#####################################################

+++F6+++

Analysis of SCF Wavefunction

Mulliken Net Atomic Charges

Atom Charge (a.u.)

----------------------------------------

1 C -0.234855

2 C -0.206450

3 C 0.401460

4 C -0.173708

5 C -0.207635

6 C -0.234533

7 H 0.306301

8 H 0.287882

9 H 0.288403

10 O -0.791920

11 C 0.309068

12 C -0.202431

13 C -0.229856

14 C 0.379516

15 C -0.207340

16 C -0.222771

17 H 0.281418

18 H 0.299799

19 Cl 0.095674

20 Cl 0.063006

21 H 0.278129

22 Cl 0.050720

23 O -0.751743

24 H 0.421866

----------------------------------------

Sum of atomic charges = 0.000000

-----------------------------------------------------------------

Cartesian Multipole Moments

-----------------------------------------------------------------

Charge (ESU x 10^10)

0.0000

Dipole Moment (Debye)

X -1.9311 Y 1.4689 Z -0.5465

Tot 2.4871

Quadrupole Moments (Debye-Ang)

XX -133.2611 XY 0.6689 YY -120.5701

XZ 0.1104 YZ -0.1613 ZZ -119.6915

Traceless Quadrupole Moments (Debye-Ang)

QXX -26.2606 QYY 11.8124 QZZ 14.4482

QXY 2.0068 QXZ 0.3313 QYZ -0.4840

Octapole Moments (Debye-Ang^2)

XXX 37.3510 XXY -12.1626 XYY 14.1516

YYY 15.0123 XXZ -13.2406 YZ 7.4845

YYZ -17.4593 XZZ 9.4465 YZZ 12.2124

ZZZ -64.6689

Traceless Octapole Moments (Debye-Ang^2)

XXX 11.7223 YYY 89.6249 ZZZ -111.7148

XXY -227.6252 XXZ 87.4977 XYY 29.4271

XYZ 112.2680 XZZ -41.1494 YYZ 24.2171

YZZ 138.0003

Hexadecapole Moments (Debye-Ang^3)

XXXX -8043.3805 XXXY -186.5283 XXYY -1565.1171

XYYY -151.8653 YYYY -1271.8292 XXXZ -112.3258

XXYZ 18.3785 XYYZ 40.2635 YYYZ 130.7194

XXZZ -1429.9774 XYZZ -57.2520 YYZZ -314.0794

XZZZ 7.6446 YZZZ 112.0502 ZZZZ -696.6367

Traceless Hexadecapole Moments (Debye-Ang^3)

XXXX -763.9485 XXXY -1781.4191 XXXZ -8895.4122

XXYY -1385.3674 XXYZ -1986.7258 XXZZ 2149.3159

XYYY 1858.1931 XYYZ 5193.9310 XYZZ -76.7740

XZZZ 3701.4812 YYYY 378.5020 YYYZ 1976.1308

YYZZ 1006.8654 YZZZ 10.5950 ZZZZ -3156.1813

-----------------------------------------------------------------

---F6---

#####################################################

# Entering drvman.exe on Tue May 25 14:59:23 2004 #

#####################################################

Calculating analytic gradient of the SCF energy

Spartan '04 Fast HF Program: (PC/x86) Release 122

Calculation started: Tue May 25 14:59:23 2004

JOBNAME.TEMP

Run type: Molecular gradient (no SCF)

Model: RHF/ABASIS

Number of shells: 71

31 S shells

37 SP shells

3 6D shells

Number of basis functions: 197

Number of electrons: 146

Number of heavy atoms: 17

Number of hydrogens: 7

Use of molecular symmetry disabled

Molecular charge: 0

Spin multiplicity: 1

Memory model: direct 13.0 Mb

Point Group = C1 Order = 1 Nsymop = 1

This system has 0 degrees of freedom

Cartesian gradient (a.u.)

Atom X Y Z

---- ------------ ------------ ------------

C 1 0.0002655 -0.0001235 -0.0000387

C 2 0.0000235 0.0000694 0.0001569

C 3 0.0007213 0.0000889 0.0003722

C 4 0.0001408 0.0000159 -0.0000606

C 5 -0.0001155 -0.0000175 -0.0000132

C 6 -0.0005662 -0.0001874 -0.0004500

H 7 0.0001396 0.0000230 -0.0001246

H 8 -0.0002743 0.0000341 0.0001103

H 9 0.0000308 0.0000061 0.0000111

O 10 -0.0004761 0.0000886 -0.0001000

C 11 -0.0004669 0.0002349 0.0004024

C 12 0.0001929 0.0000475 -0.0001048

C 13 0.0000577 -0.0000858 -0.0000451

C 14 -0.0000592 -0.0001142 -0.0000472

C 15 0.0001532 0.0001986 0.0001430

C 16 0.0000736 0.0000665 0.0000624

H 17 -0.0000118 -0.0000262 0.0000307

H 18 0.0000651 0.0000022 -0.0000579

Cl 19 0.0000718 0.0000913 -0.0001487

Cl 20 0.0001500 0.0000184 -0.0000402

H 21 -0.0000530 -0.0001781 -0.0001001

Cl 22 -0.0000776 -0.0001144 0.0000103

O 23 -0.0001281 -0.0001394 0.0001227

H 24 0.0001429 0.0000013 -0.0000907

E(HF) = -1977.0269349 a.u.

Reason for exit: Successful completion

Fast HF Program CPU Time : 000:00:12.4

Fast HF Program Wall Time: 000:00:13.3

Gradient of SCF Energy

1 2 3 4 5 6

1 0.0002655 0.0000235 0.0007213 0.0001408 -0.0001155 -0.0005662

2 -0.0001235 0.0000694 0.0000889 0.0000159 -0.0000175 -0.0001874

3 -0.0000387 0.0001569 0.0003722 -0.0000606 -0.0000132 -0.0004500

7 8 9 10 11 12

1 0.0001396 -0.0002743 0.0000308 -0.0004761 -0.0004669 0.0001929

2 0.0000230 0.0000341 0.0000061 0.0000886 0.0002349 0.0000475

3 -0.0001246 0.0001103 0.0000111 -0.0001000 0.0004024 -0.0001048

13 14 15 16 17 18

1 0.0000577 -0.0000592 0.0001532 0.0000736 -0.0000118 0.0000651

2 -0.0000858 -0.0001142 0.0001986 0.0000665 -0.0000262 0.0000022

3 -0.0000451 -0.0000472 0.0001430 0.0000624 0.0000307 -0.0000579

19 20 21 22 23 24

1 0.0000718 0.0001500 -0.0000530 -0.0000776 -0.0001281 0.0001429

2 0.0000913 0.0000184 -0.0001781 -0.0001144 -0.0001394 0.0000013

3 -0.0001487 -0.0000402 -0.0001001 0.0000103 0.0001227 -0.0000907

Max gradient component = 7.213E-004

RMS gradient = 1.892E-004

Gradient time: CPU 13.08 s wall 14047.00 s

+++F6+++

---F6---

#####################################################

# Entering optman.exe on Tue May 25 14:59:37 2004 #

#####################################################

#####################################################

# Entering fldman.exe on Tue May 25 14:59:38 2004 #

#####################################################

Requested basis set is 3-21G(d)

There are 71 shells and 197 basis functions

Applying Cartesian multipole field

Component Value

--------- -----

(2,0,0) 1.00000E-010

(0,2,0) 2.00000E-010

(0,0,2) -3.00000E-010

#####################################################

# Entering gesman.exe on Tue May 25 14:59:38 2004 #

#####################################################

Smallest overlap matrix eigenvalue = 1.34E-003

Multipole matrices computed through 2nd order

#####################################################

# Entering scfman.exe on Tue May 25 14:59:38 2004 #

#####################################################

A restricted Hartree-Fock SCF calculation will be

performed using Pulay DIIS extrapolation

SCF converges when DIIS error is below 1.0E-007

---------------------------------------

Cycle Energy DIIS Error

---------------------------------------

1 -1977.0206462500 5.00E-004

2 -1977.0269003254 5.77E-005

3 -1977.0269353684 2.27E-005

4 -1977.0269402147 1.25E-005

5 -1977.0269414984 2.55E-006

6 -1977.0269415315 1.10E-006

7 -1977.0269415707 2.80E-007

8 -1977.0269415689 9.76E-008 Convergence criterion met

---------------------------------------

SCF time: CPU 26.91 s wall 29000.00 s

#####################################################

# Entering anlman.exe on Tue May 25 15:00:08 2004 #

#####################################################

+++F6+++

Analysis of SCF Wavefunction

Mulliken Net Atomic Charges

Atom Charge (a.u.)

----------------------------------------

1 C -0.234219

2 C -0.206356

3 C 0.399699

4 C -0.173908

5 C -0.207151

6 C -0.234949

7 H 0.306453

8 H 0.288167

9 H 0.288476

10 O -0.789331

11 C 0.307603

12 C -0.201935

13 C -0.229688

14 C 0.379486

15 C -0.207503

16 C -0.222888

17 H 0.281647

18 H 0.299920

19 Cl 0.095327

20 Cl 0.062569

21 H 0.278226

22 Cl 0.050298

23 O -0.751690

24 H 0.421747

----------------------------------------

Sum of atomic charges = 0.000000

-----------------------------------------------------------------

Cartesian Multipole Moments

-----------------------------------------------------------------

Charge (ESU x 10^10)

0.0000

Dipole Moment (Debye)

X -1.9231 Y 1.4491 Z -0.5369

Tot 2.4671

Quadrupole Moments (Debye-Ang)

XX -133.2200 XY 0.6311 YY -120.6743

XZ 0.0864 YZ -0.1168 ZZ -119.6590

Traceless Quadrupole Moments (Debye-Ang)

QXX -26.1067 QYY 11.5305 QZZ 14.5762

QXY 1.8933 QXZ 0.2591 QYZ -0.3504

Octapole Moments (Debye-Ang^2)

XXX 37.7104 XXY -12.5049 XYY 14.4752

YYY 13.6709 XXZ -13.1435 YZ 7.2997

YYZ -17.5157 XZZ 9.5467 YZZ 12.0386

ZZZ -64.7142

Traceless Octapole Moments (Debye-Ang^2)

XXX 10.0658 YYY 86.2215 ZZZ -112.3520

XXY -227.1871 XXZ 88.9680 XYY 31.9309

XYZ 109.4951 XZZ -41.9967 YYZ 23.3840

YZZ 140.9656

Hexadecapole Moments (Debye-Ang^3)

XXXX -8016.7136 XXXY -187.0668 XXYY -1562.2178

XYYY -153.0703 YYYY -1272.6564 XXXZ -109.9981

XXYZ 18.9587 XYYZ 41.5591 YYYZ 132.0530

XXZZ -1427.0004 XYZZ -57.1280 YYZZ -315.9981

XZZZ 11.5593 YZZZ 112.7603 ZZZZ -706.3228

Traceless Hexadecapole Moments (Debye-Ang^3)

XXXX -676.1947 XXXY -1765.0857 XXXZ -8990.2172

XXYY -1499.1858 XXYZ -1954.5127 XXZZ 2175.3805

XYYY 1804.5501 XYYZ 5216.9052 XYZZ -39.4644

XZZZ 3773.3120 YYYY 494.4572 YYYZ 2030.0398

YYZZ 1004.7286 YZZZ -75.5271 ZZZZ -3180.1091

-----------------------------------------------------------------

---F6---

#####################################################

# Entering drvman.exe on Tue May 25 15:00:09 2004 #

#####################################################

Calculating analytic gradient of the SCF energy

Spartan '04 Fast HF Program: (PC/x86) Release 122

Calculation started: Tue May 25 15:00:10 2004

JOBNAME.TEMP

Run type: Molecular gradient (no SCF)

Model: RHF/ABASIS

Number of shells: 71

31 S shells

37 SP shells

3 6D shells

Number of basis functions: 197

Number of electrons: 146

Number of heavy atoms: 17

Number of hydrogens: 7

Use of molecular symmetry disabled

Molecular charge: 0

Spin multiplicity: 1

Memory model: direct 13.0 Mb

Point Group = C1 Order = 1 Nsymop = 1

This system has 0 degrees of freedom

Cartesian gradient (a.u.)

Atom X Y Z

---- ------------ ------------ ------------

C 1 -0.0002668 0.0001330 -0.0000551

C 2 0.0001537 0.0000338 -0.0002115

C 3 -0.0001963 0.0002159 0.0003033

C 4 -0.0000026 -0.0002288 0.0002870

C 5 -0.0001319 0.0001583 -0.0001018

C 6 0.0002789 0.0000067 -0.0001272

H 7 -0.0000956 -0.0001180 0.0001542

H 8 0.0001139 0.0000657 -0.0001148

H 9 -0.0000621 0.0002165 -0.0001889

O 10 0.0002042 -0.0002101 -0.0004159

C 11 0.0001136 0.0001838 0.0002423

C 12 -0.0001024 -0.0003665 -0.0001834

C 13 0.0001064 -0.0000240 -0.0002335

C 14 0.0004159 0.0000374 -0.0001550

C 15 -0.0003598 0.0000424 0.0002025

C 16 -0.0004205 -0.0000585 0.0000874

H 17 -0.0001960 0.0000765 0.0001078

H 18 0.0002799 -0.0001546 -0.0001751

Cl 19 0.0000534 -0.0004545 0.0005228

Cl 20 -0.0002738 0.0001418 -0.0000463

H 21 -0.0000443 0.0000107 -0.0000008

Cl 22 0.0002960 0.0000617 0.0000096

O 23 0.0003634 0.0002267 0.0000140

H 24 -0.0002272 0.0000041 0.0000782

E(HF) = -1977.0269416 a.u.

Reason for exit: Successful completion

Fast HF Program CPU Time : 000:00:12.5

Fast HF Program Wall Time: 000:00:13.9

Gradient of SCF Energy

1 2 3 4 5 6

1 -0.0002668 0.0001537 -0.0001963 -0.0000026 -0.0001319 0.0002789

2 0.0001330 0.0000338 0.0002159 -0.0002288 0.0001583 0.0000067

3 -0.0000551 -0.0002115 0.0003033 0.0002870 -0.0001018 -0.0001272

7 8 9 10 11 12

1 -0.0000956 0.0001139 -0.0000621 0.0002042 0.0001136 -0.0001024

2 -0.0001180 0.0000657 0.0002165 -0.0002101 0.0001838 -0.0003665

3 0.0001542 -0.0001148 -0.0001889 -0.0004159 0.0002423 -0.0001834

13 14 15 16 17 18

1 0.0001064 0.0004159 -0.0003598 -0.0004205 -0.0001960 0.0002799

2 -0.0000240 0.0000374 0.0000424 -0.0000585 0.0000765 -0.0001546

3 -0.0002335 -0.0001550 0.0002025 0.0000874 0.0001078 -0.0001751

19 20 21 22 23 24

1 0.0000534 -0.0002738 -0.0000443 0.0002960 0.0003634 -0.0002272

2 -0.0004545 0.0001418 0.0000107 0.0000617 0.0002267 0.0000041

3 0.0005228 -0.0000463 -0.0000008 0.0000096 0.0000140 0.0000782

Max gradient component = 5.228E-004

RMS gradient = 2.060E-004

Gradient time: CPU 13.23 s wall 14687.00 s

+++F6+++

---F6---

#####################################################

# Entering optman.exe on Tue May 25 15:00:25 2004 #

#####################################################

#####################################################

# Entering fldman.exe on Tue May 25 15:00:26 2004 #

#####################################################

Requested basis set is 3-21G(d)

There are 71 shells and 197 basis functions

Applying Cartesian multipole field

Component Value

--------- -----

(2,0,0) 1.00000E-010

(0,2,0) 2.00000E-010

(0,0,2) -3.00000E-010

#####################################################

# Entering gesman.exe on Tue May 25 15:00:26 2004 #

#####################################################

Smallest overlap matrix eigenvalue = 1.33E-003

Multipole matrices computed through 2nd order

#####################################################

# Entering scfman.exe on Tue May 25 15:00:26 2004 #

#####################################################

A restricted Hartree-Fock SCF calculation will be

performed using Pulay DIIS extrapolation

SCF converges when DIIS error is below 1.0E-007

---------------------------------------

Cycle Energy DIIS Error

---------------------------------------

1 -1977.0312028435 2.57E-004

2 -1977.0269312275 3.10E-005

3 -1977.0269426686 1.08E-005

4 -1977.0269443782 3.70E-006

5 -1977.0269445217 1.22E-006

6 -1977.0269445444 4.26E-007

7 -1977.0269445406 1.44E-007

8 -1977.0269445317 4.55E-008 Convergence criterion met

---------------------------------------

SCF time: CPU 25.33 s wall 28172.00 s

#####################################################

# Entering anlman.exe on Tue May 25 15:00:55 2004 #

#####################################################

+++F6+++

Analysis of SCF Wavefunction

Mulliken Net Atomic Charges

Atom Charge (a.u.)

----------------------------------------

1 C -0.234668

2 C -0.206377

3 C 0.399816

4 C -0.173774

5 C -0.207471

6 C -0.234654

7 H 0.306369

8 H 0.288057

9 H 0.288425

10 O -0.789493

11 C 0.306986

12 C -0.202243

13 C -0.229163

14 C 0.379698

15 C -0.207345

16 C -0.222907

17 H 0.281569

18 H 0.299817

19 Cl 0.095764

20 Cl 0.062784

21 H 0.278224

22 Cl 0.050767

23 O -0.751960

24 H 0.421779

----------------------------------------

Sum of atomic charges = 0.000000

-----------------------------------------------------------------

Cartesian Multipole Moments

-----------------------------------------------------------------

Charge (ESU x 10^10)

0.0000

Dipole Moment (Debye)

X -1.9104 Y 1.4401 Z -0.5290

Tot 2.4502

Quadrupole Moments (Debye-Ang)

XX -133.1762 XY 0.6443 YY -120.6922

XZ 0.1048 YZ -0.0797 ZZ -119.6157

Traceless Quadrupole Moments (Debye-Ang)

QXX -26.0446 QYY 11.4075 QZZ 14.6371

QXY 1.9330 QXZ 0.3143 QYZ -0.2392

Octapole Moments (Debye-Ang^2)

XXX 37.8833 XXY -12.4665 XYY 14.6155

YYY 13.1968 XXZ -13.0751 YZ 7.2792

YYZ -17.4297 XZZ 9.6004 YZZ 12.0701

ZZZ -64.5409

Traceless Octapole Moments (Debye-Ang^2)

XXX 9.3559 YYY 82.7486 ZZZ -112.7025

XXY -225.3994 XXZ 89.0103 XYY 32.9354

XYZ 109.1884 XZZ -42.2913 YYZ 23.6922

YZZ 142.6508

Hexadecapole Moments (Debye-Ang^3)

XXXX -8013.2897 XXXY -188.2856 XXYY -1560.7047

XYYY -154.7449 YYYY -1267.1865 XXXZ -110.8987

XXYZ 19.3290 XYYZ 41.3632 YYYZ 132.2530

XXZZ -1426.9052 XYZZ -57.5065 YYZZ -315.8704

XZZZ 11.1796 YZZZ 112.6923 ZZZZ -710.7587

Traceless Hexadecapole Moments (Debye-Ang^3)

XXXX -698.2086 XXXY -1745.8238 XXXZ -9018.3436

XXYY -1498.6658 XXYZ -1924.1831 XXZZ 2196.8744

XYYY 1775.9508 XYYZ 5218.4726 XYZZ -30.1270

XZZZ 3799.8710 YYYY 500.2069 YYYZ 2025.3756

YYZZ 998.4588 YZZZ -101.1925 ZZZZ -3195.3332

-----------------------------------------------------------------

---F6---

#####################################################

# Entering drvman.exe on Tue May 25 15:00:56 2004 #

#####################################################

Calculating analytic gradient of the SCF energy

Spartan '04 Fast HF Program: (PC/x86) Release 122

Calculation started: Tue May 25 15:00:56 2004

JOBNAME.TEMP

Run type: Molecular gradient (no SCF)

Model: RHF/ABASIS

Number of shells: 71

31 S shells

37 SP shells

3 6D shells

Number of basis functions: 197

Number of electrons: 146

Number of heavy atoms: 17

Number of hydrogens: 7

Use of molecular symmetry disabled

Molecular charge: 0

Spin multiplicity: 1

Memory model: direct 13.0 Mb

Point Group = C1 Order = 1 Nsymop = 1

This system has 0 degrees of freedom

Cartesian gradient (a.u.)

Atom X Y Z

---- ------------ ------------ ------------

C 1 0.0001239 0.0000207 -0.0000595

C 2 -0.0001784 -0.0000106 -0.0000513

C 3 -0.0000640 0.0001735 0.0003737

C 4 -0.0001099 0.0001159 -0.0000345

C 5 0.0000406 -0.0000284 0.0000642

C 6 0.0003075 -0.0002958 -0.0000008

H 7 -0.0000076 0.0000421 -0.0000567

H 8 -0.0000251 -0.0000591 0.0000872

H 9 -0.0000250 -0.0000504 0.0000914

O 10 -0.0000626 -0.0000328 -0.0004882

C 11 0.0001329 -0.0001035 0.0000851

C 12 -0.0000407 0.0000423 0.0000133

C 13 -0.0002829 -0.0001505 -0.0000858

C 14 -0.0003838 -0.0000239 0.0000648

C 15 0.0004120 0.0001730 -0.0000236

C 16 0.0004318 0.0000051 -0.0001661

H 17 0.0000699 -0.0001445 -0.0000873

H 18 -0.0001694 0.0001806 0.0001567

Cl 19 -0.0000181 0.0000519 -0.0000610

Cl 20 -0.0000476 0.0000004 0.0000164

H 21 0.0000574 -0.0000685 -0.0000541

Cl 22 -0.0000599 -0.0000448 0.0000767

O 23 -0.0000890 0.0002406 0.0001597

H 24 -0.0000122 -0.0000333 -0.0000202

E(HF) = -1977.0269445 a.u.

Reason for exit: Successful completion

Fast HF Program CPU Time : 000:00:12.5

Fast HF Program Wall Time: 000:00:12.8

Gradient of SCF Energy

1 2 3 4 5 6

1 0.0001239 -0.0001784 -0.0000640 -0.0001099 0.0000406 0.0003075

2 0.0000207 -0.0000106 0.0001735 0.0001159 -0.0000284 -0.0002958

3 -0.0000595 -0.0000513 0.0003737 -0.0000345 0.0000642 -0.0000008

7 8 9 10 11 12

1 -0.0000076 -0.0000251 -0.0000250 -0.0000626 0.0001329 -0.0000407

2 0.0000421 -0.0000591 -0.0000504 -0.0000328 -0.0001035 0.0000423

3 -0.0000567 0.0000872 0.0000914 -0.0004882 0.0000851 0.0000133

13 14 15 16 17 18

1 -0.0002829 -0.0003838 0.0004120 0.0004318 0.0000699 -0.0001694

2 -0.0001505 -0.0000239 0.0001730 0.0000051 -0.0001445 0.0001806

3 -0.0000858 0.0000648 -0.0000236 -0.0001661 -0.0000873 0.0001567

19 20 21 22 23 24

1 -0.0000181 -0.0000476 0.0000574 -0.0000599 -0.0000890 -0.0000122

2 0.0000519 0.0000004 -0.0000685 -0.0000448 0.0002406 -0.0000333

3 -0.0000610 0.0000164 -0.0000541 0.0000767 0.0001597 -0.0000202

Max gradient component = 4.882E-004

RMS gradient = 1.525E-004

Gradient time: CPU 13.19 s wall 13594.00 s

+++F6+++

---F6---

#####################################################

# Entering optman.exe on Tue May 25 15:01:10 2004 #

#####################################################

#####################################################

# Entering fldman.exe on Tue May 25 15:01:10 2004 #

#####################################################

Requested basis set is 3-21G(d)

There are 71 shells and 197 basis functions

Applying Cartesian multipole field

Component Value

--------- -----

(2,0,0) 1.00000E-010

(0,2,0) 2.00000E-010

(0,0,2) -3.00000E-010

#####################################################

# Entering gesman.exe on Tue May 25 15:01:11 2004 #

#####################################################

Smallest overlap matrix eigenvalue = 1.33E-003

Multipole matrices computed through 2nd order

#####################################################

# Entering scfman.exe on Tue May 25 15:01:11 2004 #

#####################################################

A restricted Hartree-Fock SCF calculation will be

performed using Pulay DIIS extrapolation

SCF converges when DIIS error is below 1.0E-007

---------------------------------------

Cycle Energy DIIS Error

---------------------------------------

1 -1977.0261483505 2.04E-004

2 -1977.0269347808 2.51E-005

3 -1977.0269425398 8.93E-006

4 -1977.0269436985 3.30E-006

5 -1977.0269437832 9.85E-007

6 -1977.0269437839 2.69E-007

7 -1977.0269438169 9.60E-008 Convergence criterion met

---------------------------------------

SCF time: CPU 23.20 s wall 25453.00 s

#####################################################

# Entering anlman.exe on Tue May 25 15:01:38 2004 #

#####################################################

+++F6+++

Analysis of SCF Wavefunction

Mulliken Net Atomic Charges

Atom Charge (a.u.)

----------------------------------------

1 C -0.234760

2 C -0.206413

3 C 0.399673

4 C -0.173776

5 C -0.207465

6 C -0.234424

7 H 0.306349

8 H 0.287943

9 H 0.288431

10 O -0.789292

11 C 0.306894

12 C -0.201955

13 C -0.228988

14 C 0.379598

15 C -0.207425

16 C -0.223004

17 H 0.281565

18 H 0.299852

19 Cl 0.095752

20 Cl 0.062800

21 H 0.278263

22 Cl 0.050538

23 O -0.751821

24 H 0.421664

----------------------------------------

Sum of atomic charges = 0.000000

-----------------------------------------------------------------

Cartesian Multipole Moments

-----------------------------------------------------------------

Charge (ESU x 10^10)

0.0000

Dipole Moment (Debye)

X -1.9068 Y 1.4362 Z -0.5276

Tot 2.4448

Quadrupole Moments (Debye-Ang)

XX -133.1907 XY 0.6395 YY -120.7172

XZ 0.1192 YZ -0.0526 ZZ -119.5726

Traceless Quadrupole Moments (Debye-Ang)

QXX -26.0915 QYY 11.3289 QZZ 14.7627

QXY 1.9186 QXZ 0.3575 QYZ -0.1577

Octapole Moments (Debye-Ang^2)

XXX 37.7798 XXY -12.4946 XYY 14.6641

YYY 12.9040 XXZ -13.0786 YZ 7.2981

YYZ -17.3883 XZZ 9.5812 YZZ 12.1294

ZZZ -64.4855

Traceless Octapole Moments (Debye-Ang^2)

XXX 8.4711 YYY 80.7112 ZZZ -112.7105

XXY -225.0352 XXZ 88.6783 XYY 33.8859

XYZ 109.4722 XZZ -42.3570 YYZ 24.0321

YZZ 144.3240

Hexadecapole Moments (Debye-Ang^3)

XXXX -8016.5595 XXXY -189.0190 XXYY -1560.0860

XYYY -155.4380 YYYY -1261.8514 XXXZ -112.2983

XXYZ 19.4802 XYYZ 41.0840 YYYZ 132.4083

XXZZ -1427.6377 XYZZ -57.6204 YYZZ -315.6788

XZZZ 10.4683 YZZZ 112.5627 ZZZZ -715.1767

Traceless Hexadecapole Moments (Debye-Ang^3)

XXXX -756.7960 XXXY -1753.5130 XXXZ -9057.7527

XXYY -1481.7128 XXYZ -1912.9022 XXZZ 2238.5088

XYYY 1772.4941 XYYZ 5225.0112 XYZZ -18.9811

XZZZ 3832.7415 YYYY 487.5278 YYYZ 2027.8877

YYZZ 994.1850 YZZZ -114.9854 ZZZZ -3232.6938

-----------------------------------------------------------------

---F6---

#####################################################

# Entering drvman.exe on Tue May 25 15:01:38 2004 #

#####################################################

Calculating analytic gradient of the SCF energy

Spartan '04 Fast HF Program: (PC/x86) Release 122

Calculation started: Tue May 25 15:01:39 2004

JOBNAME.TEMP

Run type: Molecular gradient (no SCF)

Model: RHF/ABASIS

Number of shells: 71

31 S shells

37 SP shells

3 6D shells

Number of basis functions: 197

Number of electrons: 146

Number of heavy atoms: 17

Number of hydrogens: 7

Use of molecular symmetry disabled

Molecular charge: 0

Spin multiplicity: 1

Memory model: direct 13.0 Mb

Point Group = C1 Order = 1 Nsymop = 1

This system has 0 degrees of freedom

Cartesian gradient (a.u.)

Atom X Y Z

---- ------------ ------------ ------------

C 1 -0.0000073 -0.0000138 0.0000287

C 2 0.0000206 -0.0000662 0.0000620

C 3 0.0001479 0.0002551 0.0001929

C 4 0.0000494 0.0000197 -0.0000124

C 5 0.0000275 0.0000008 -0.0000033

C 6 -0.0002109 -0.0001556 -0.0001288

H 7 0.0000043 -0.0000141 0.0000124

H 8 0.0000236 0.0000137 -0.0000316

H 9 -0.0000102 0.0000155 -0.0000109

O 10 -0.0001145 -0.0000938 -0.0002323

C 11 -0.0000265 0.0000670 0.0001889

C 12 -0.0000649 -0.0001138 -0.0000610

C 13 -0.0000186 -0.0000632 -0.0000783

C 14 -0.0000608 -0.0000482 -0.0000579

C 15 -0.0000192 0.0000898 0.0001000

C 16 -0.0000453 0.0000204 0.0000046

H 17 0.0000014 -0.0000475 0.0000127

H 18 0.0000476 0.0000068 -0.0000090

Cl 19 -0.0000061 0.0000449 -0.0000640

Cl 20 0.0000351 -0.0000266 0.0000186

H 21 0.0000157 -0.0000683 -0.0000449

Cl 22 0.0002047 0.0000489 0.0000354

O 23 0.0000843 0.0001529 0.0000775

H 24 -0.0000776 -0.0000243 0.0000007

E(HF) = -1977.0269438 a.u.

Reason for exit: Successful completion

Fast HF Program CPU Time : 000:00:12.5

Fast HF Program Wall Time: 000:00:13.6

Gradient of SCF Energy

1 2 3 4 5 6

1 -0.0000073 0.0000206 0.0001479 0.0000494 0.0000275 -0.0002109

2 -0.0000138 -0.0000662 0.0002551 0.0000197 0.0000008 -0.0001556

3 0.0000287 0.0000620 0.0001929 -0.0000124 -0.0000033 -0.0001288

7 8 9 10 11 12

1 0.0000043 0.0000236 -0.0000102 -0.0001145 -0.0000265 -0.0000649

2 -0.0000141 0.0000137 0.0000155 -0.0000938 0.0000670 -0.0001138

3 0.0000124 -0.0000316 -0.0000109 -0.0002323 0.0001889 -0.0000610

13 14 15 16 17 18

1 -0.0000186 -0.0000608 -0.0000192 -0.0000453 0.0000014 0.0000476

2 -0.0000632 -0.0000482 0.0000898 0.0000204 -0.0000475 0.0000068

3 -0.0000783 -0.0000579 0.0001000 0.0000046 0.0000127 -0.0000090

19 20 21 22 23 24

1 -0.0000061 0.0000351 0.0000157 0.0002047 0.0000843 -0.0000776

2 0.0000449 -0.0000266 -0.0000683 0.0000489 0.0001529 -0.0000243

3 -0.0000640 0.0000186 -0.0000449 0.0000354 0.0000775 0.0000007

Max gradient component = 2.551E-004

RMS gradient = 8.439E-005

Gradient time: CPU 13.16 s wall 14390.00 s

+++F6+++

---F6---

#####################################################

# Entering optman.exe on Tue May 25 15:01:53 2004 #

#####################################################

#####################################################

# Entering fldman.exe on Tue May 25 15:01:53 2004 #

#####################################################

Requested basis set is 3-21G(d)

There are 71 shells and 197 basis functions

Applying Cartesian multipole field

Component Value

--------- -----

(2,0,0) 1.00000E-010

(0,2,0) 2.00000E-010

(0,0,2) -3.00000E-010

#####################################################

# Entering gesman.exe on Tue May 25 15:01:54 2004 #

#####################################################

Smallest overlap matrix eigenvalue = 1.34E-003

Multipole matrices computed through 2nd order

#####################################################

# Entering scfman.exe on Tue May 25 15:01:54 2004 #

#####################################################

A restricted Hartree-Fock SCF calculation will be

performed using Pulay DIIS extrapolation

SCF converges when DIIS error is below 1.0E-007

---------------------------------------

Cycle Energy DIIS Error

---------------------------------------

1 -1977.0263130387 1.11E-004

2 -1977.0269418374 1.32E-005

3 -1977.0269439228 4.68E-006

4 -1977.0269442084 2.18E-006

5 -1977.0269442586 5.48E-007

6 -1977.0269442207 1.69E-007

7 -1977.0269442125 5.97E-008 Convergence criterion met

---------------------------------------

SCF time: CPU 22.52 s wall 25328.00 s

#####################################################

# Entering anlman.exe on Tue May 25 15:02:20 2004 #

#####################################################

+++F6+++

Analysis of SCF Wavefunction

Mulliken Net Atomic Charges

Atom Charge (a.u.)

----------------------------------------

1 C -0.234689

2 C -0.206384

3 C 0.399486

4 C -0.173792

5 C -0.207430

6 C -0.234452

7 H 0.306348

8 H 0.287962

9 H 0.288435

10 O -0.789020

11 C 0.306727

12 C -0.201978

13 C -0.228919

14 C 0.379473

15 C -0.207442

16 C -0.222998

17 H 0.281582

18 H 0.299858

19 Cl 0.095713

20 Cl 0.062744

21 H 0.278276

22 Cl 0.050616

23 O -0.751745

24 H 0.421628

----------------------------------------

Sum of atomic charges = 0.000000

-----------------------------------------------------------------

Cartesian Multipole Moments

-----------------------------------------------------------------

Charge (ESU x 10^10)

0.0000

Dipole Moment (Debye)

X -1.9022 Y 1.4324 Z -0.5244

Tot 2.4383

Quadrupole Moments (Debye-Ang)

XX -133.1756 XY 0.6379 YY -120.7348

XZ 0.1199 YZ -0.0382 ZZ -119.5619

Traceless Quadrupole Moments (Debye-Ang)

QXX -26.0544 QYY 11.2677 QZZ 14.7867

QXY 1.9138 QXZ 0.3597 QYZ -0.1147

Octapole Moments (Debye-Ang^2)

XXX 37.9098 XXY -12.5292 XYY 14.7415

YYY 12.6252 XXZ -13.0515 YZ 7.2689

YYZ -17.3810 XZZ 9.6156 YZZ 12.1146

ZZZ -64.4539

Traceless Octapole Moments (Debye-Ang^2)

XXX 8.2453 YYY 79.4827 ZZZ -112.8313

XXY -224.5697 XXZ 88.8867 XYY 34.3216

XYZ 109.0340 XZZ -42.5669 YYZ 23.9446

YZZ 145.0871

Hexadecapole Moments (Debye-Ang^3)

XXXX -8011.9341 XXXY -189.2085 XXYY -1559.3718

XYYY -155.8395 YYYY -1260.9462 XXXZ -112.1373

XXYZ 19.6517 XYYZ 41.2403 YYYZ 132.6994

XXZZ -1427.2225 XYZZ -57.6413 YYZZ -315.9756

XZZZ 10.9641 YZZZ 112.7011 ZZZZ -717.6684

Traceless Hexadecapole Moments (Debye-Ang^3)

XXXX -746.7221 XXXY -1745.8764 XXXZ -9077.4364

XXYY -1498.7763 XXYZ -1901.8416 XXZZ 2245.4984

XYYY 1757.8755 XYYZ 5229.2262 XYZZ -11.9990

XZZZ 3848.2102 YYYY 505.8773 YYYZ 2037.6398

YYZZ 992.8989 YZZZ -135.7982 ZZZZ -3238.3974

-----------------------------------------------------------------

---F6---

#####################################################

# Entering drvman.exe on Tue May 25 15:02:21 2004 #

#####################################################

Calculating analytic gradient of the SCF energy

Spartan '04 Fast HF Program: (PC/x86) Release 122

Calculation started: Tue May 25 15:02:21 2004

JOBNAME.TEMP

Run type: Molecular gradient (no SCF)

Model: RHF/ABASIS

Number of shells: 71

31 S shells

37 SP shells

3 6D shells

Number of basis functions: 197

Number of electrons: 146

Number of heavy atoms: 17

Number of hydrogens: 7

Use of molecular symmetry disabled

Molecular charge: 0

Spin multiplicity: 1

Memory model: direct 13.0 Mb

Point Group = C1 Order = 1 Nsymop = 1

This system has 0 degrees of freedom

Cartesian gradient (a.u.)

Atom X Y Z

---- ------------ ------------ ------------

C 1 0.0000297 -0.0000026 0.0000118

C 2 -0.0000463 -0.0000475 0.0000103

C 3 0.0000737 0.0002032 0.0002469

C 4 -0.0000416 0.0000607 -0.0000274

C 5 0.0000476 0.0000024 0.0000040

C 6 0.0000037 -0.0001574 -0.0001082

H 7 0.0000014 -0.0000023 0.0000079

H 8 -0.0000100 -0.0000101 0.0000040

H 9 -0.0000207 0.0000092 0.0000013

O 10 -0.0000008 -0.0000255 -0.0003093

C 11 -0.0000443 0.0000153 0.0001672

C 12 -0.0000065 -0.0000613 -0.0000406

C 13 -0.0000405 -0.0000026 -0.0000385

C 14 -0.0000348 -0.0000165 -0.0000528

C 15 0.0000533 0.0000658 0.0000434

C 16 -0.0000562 0.0000398 0.0000050

H 17 0.0000017 -0.0000408 0.0000080

H 18 0.0000267 -0.0000060 -0.0000005

Cl 19 0.0000039 -0.0000203 0.0000156

Cl 20 -0.0000325 0.0000007 0.0000089

H 21 0.0000087 -0.0000186 -0.0000115

Cl 22 0.0000963 -0.0000002 0.0000435

O 23 0.0000515 0.0000392 0.0000093

H 24 -0.0000641 -0.0000248 0.0000016

E(HF) = -1977.0269442 a.u.

Reason for exit: Successful completion

Fast HF Program CPU Time : 000:00:12.4

Fast HF Program Wall Time: 000:00:14.1

Gradient of SCF Energy

1 2 3 4 5 6

1 0.0000297 -0.0000463 0.0000737 -0.0000416 0.0000476 0.0000037

2 -0.0000026 -0.0000475 0.0002032 0.0000607 0.0000024 -0.0001574

3 0.0000118 0.0000103 0.0002469 -0.0000274 0.0000040 -0.0001082

7 8 9 10 11 12

1 0.0000014 -0.0000100 -0.0000207 -0.0000008 -0.0000443 -0.0000065

2 -0.0000023 -0.0000101 0.0000092 -0.0000255 0.0000153 -0.0000613

3 0.0000079 0.0000040 0.0000013 -0.0003093 0.0001672 -0.0000406

13 14 15 16 17 18

1 -0.0000405 -0.0000348 0.0000533 -0.0000562 0.0000017 0.0000267

2 -0.0000026 -0.0000165 0.0000658 0.0000398 -0.0000408 -0.0000060

3 -0.0000385 -0.0000528 0.0000434 0.0000050 0.0000080 -0.0000005

19 20 21 22 23 24

1 0.0000039 -0.0000325 0.0000087 0.0000963 0.0000515 -0.0000641

2 -0.0000203 0.0000007 -0.0000186 -0.0000002 0.0000392 -0.0000248

3 0.0000156 0.0000089 -0.0000115 0.0000435 0.0000093 0.0000016

Max gradient component = 3.093E-004

RMS gradient = 6.843E-005

Gradient time: CPU 13.08 s wall 14891.00 s

+++F6+++

---F6---

#####################################################

# Entering optman.exe on Tue May 25 15:02:36 2004 #

#####################################################

#####################################################

# Entering anlman.exe on Tue May 25 15:02:37 2004 #

#####################################################

+++F6+++

Analysis of SCF Wavefunction

Mulliken Net Atomic Charges

Atom Charge (a.u.)

----------------------------------------

1 C -0.234689

2 C -0.206384

3 C 0.399486

4 C -0.173792

5 C -0.207430

6 C -0.234452

7 H 0.306348

8 H 0.287962

9 H 0.288435

10 O -0.789020

11 C 0.306727

12 C -0.201978

13 C -0.228919

14 C 0.379473

15 C -0.207442

16 C -0.222998

17 H 0.281582

18 H 0.299858

19 Cl 0.095713

20 Cl 0.062744

21 H 0.278276

22 Cl 0.050616

23 O -0.751745

24 H 0.421628

----------------------------------------

Sum of atomic charges = 0.000000

-----------------------------------------------------------------

Cartesian Multipole Moments

-----------------------------------------------------------------

Charge (ESU x 10^10)

0.0000

Dipole Moment (Debye)

X -1.9022 Y 1.4324 Z -0.5244

Tot 2.4383

Quadrupole Moments (Debye-Ang)

XX -133.1756 XY 0.6379 YY -120.7348

XZ 0.1199 YZ -0.0382 ZZ -119.5619

Traceless Quadrupole Moments (Debye-Ang)

QXX -26.0544 QYY 11.2677 QZZ 14.7867

QXY 1.9138 QXZ 0.3597 QYZ -0.1147

Octapole Moments (Debye-Ang^2)

XXX 37.9098 XXY -12.5292 XYY 14.7415

YYY 12.6252 XXZ -13.0515 YZ 7.2689

YYZ -17.3810 XZZ 9.6156 YZZ 12.1146

ZZZ -64.4539

Traceless Octapole Moments (Debye-Ang^2)

XXX 8.2453 YYY 79.4827 ZZZ -112.8313

XXY -224.5697 XXZ 88.8867 XYY 34.3216

XYZ 109.0340 XZZ -42.5669 YYZ 23.9446

YZZ 145.0871

Hexadecapole Moments (Debye-Ang^3)

XXXX -8011.9341 XXXY -189.2085 XXYY -1559.3718

XYYY -155.8395 YYYY -1260.9462 XXXZ -112.1373

XXYZ 19.6517 XYYZ 41.2403 YYYZ 132.6994

XXZZ -1427.2225 XYZZ -57.6413 YYZZ -315.9756

XZZZ 10.9641 YZZZ 112.7011 ZZZZ -717.6684

Traceless Hexadecapole Moments (Debye-Ang^3)

XXXX -746.7221 XXXY -1745.8764 XXXZ -9077.4364

XXYY -1498.7763 XXYZ -1901.8416 XXZZ 2245.4984

XYYY 1757.8755 XYYZ 5229.2262 XYZZ -11.9990

XZZZ 3848.2102 YYYY 505.8773 YYYZ 2037.6398

YYZZ 992.8989 YZZZ -135.7982 ZZZZ -3238.3974

-----------------------------------------------------------------

---F6---

Total job wall time: 3.5e+005 s

Spartan '04 Mechanics Program: (PC/x86) Release 120

Reading coordinates from previous archive

Adjusted 5 (out of 72) low frequency modes

Reason for exit: Successful completion

QmMm CPU Time : 000:00:00.2

QmMm Wall Time: 000:00:00.2

Spartan '04 Quantum Mechanics Program: (PC/x86) Release 120

Job type: Geometry optimization.

Method: RHF

Basis set: 3-21G(*)

Number of shells: 71

Number of basis functions: 197

SCF model:

A restricted Hartree-Fock SCF calculation will be

performed using Pulay DIIS extrapolation

Optimization:

Step Energy Max Grad. Max Dist.

1 -1976.9979263 0.049570 0.096786

2 -1977.0218055 0.015506 0.202424

3 -1977.0260576 0.010627 0.215102

4 -1977.0265769 0.009295 0.095292

5 -1977.0257044 0.010666 0.059539

6 -1977.0269290 0.002068 0.011377

7 -1977.0269612 0.001769 0.012908

8 -1977.0269899 0.000646 0.023390

9 -1977.0270113 0.001028 0.015826

10 -1977.0270260 0.001372 0.030069

11 -1977.0270479 0.001159 0.062760

12 -1977.0270725 0.000519 0.009743

13 -1977.0270761 0.000335 0.007244

14 -1977.0270774 0.000393 0.012392

15 -1977.0270780 0.000363 0.016476

16 -1977.0270774 0.000169 0.004312

Reason for exit: Sucessful completion

Quantum Mechanics Program CPU Time : 000:32:45.8

Quantum Mechanics Program Wall Time: 000:14:12.7

Spartan '04 Semi-Empirical Program: (PC/x86) Release 120

Semi-empirical Property Calculation

M001

Guess from Archive

Energy Due to Solvation

Solvation Energy SM5.4/A -3.905

Memory Used: 2.465 Mb

Reason for exit: Successful completion

Semi-Empirical Program CPU Time : 000:00:00.3

Semi-Empirical Program Wall Time: 000:00:00.3

Spartan '04 Properties Program: (PC/x86) Release 120

Reason for exit: Successful completion

Properties Program CPU Time : 000:00:04.6

Properties Program Wall Time: 000:00:04.6

Spartan '04 CONFORMER (ENERGY PROFILE) PROGRAM: (PC/x86) WIN build 121

Job run on machine: <not-available>

Submitted to computational module(s): Thu May 20 13:56:38 2004

Dihedral Move : C2 - O1 - C7 - C9 [15] 90.000000 .. 20.000000

1 ) 90.00 -1240343.64507834 Failed

!!! Error inside backend run:

Begin noisy output

---------------------------

Spartan '04 Mechanics Program: (PC/x86) Release 121

Adjusted 6 (out of 72) low frequency modes

Reason for exit: Successful completion

QmMm CPU Time : 000:00:00.1

QmMm Wall Time: 000:00:00.6

Spartan '04 Quantum Mechanics Program: (PC/x86) Release 121

Job type: Geometry optimization.

Method: RHF

Basis set: 3-21G(*)

Number of shells: 71

Number of basis functions: 197
